# Metabolic Profile and Pathological Alterations in the Muscle of Patients with Early-Stage Amyotrophic Lateral Sclerosis

**DOI:** 10.3390/biomedicines10061307

**Published:** 2022-06-02

**Authors:** Débora Lanznaster, Clément Bruno, Jérôme Bourgeais, Patrick Emond, Ilyess Zemmoura, Antoine Lefèvre, Pascal Reynier, Sébastien Eymieux, Emmanuelle Blanchard, Patrick Vourc’h, Christian R. Andres, Salah Eddine Bakkouche, Olivier Herault, Luc Favard, Philippe Corcia, Hélène Blasco

**Affiliations:** 1 UMR 1253, iBrain, Université de Tours, INSERM, 37000 Tours, France; clement.bruno@etu.univ-tours.fr (C.B.); patrick.emond@univ-tours.fr (P.E.); ilyess.zemmoura@univ-tours.fr (I.Z.); antoine.lefevre@univ-tours.fr (A.L.); patrick.vourch@univ-tours.fr (P.V.); christian.andres@univ-tours.fr (C.R.A.); philippe.corcia@univ-tours.fr (P.C.); helene.blasco@univ-tours.fr (H.B.); 2 Service de Biochimie et Biologie Moléculaire, CHU de Tours, 37000 Tours, France; 3 CNRS ERL7001, EA 7501 GICC, Université de Tours, 37000 Tours, France; j.bourgeais@chu-tours.fr (J.B.); olivier.herault@univ-tours.fr (O.H.); 4 Service de Médecine Nucléaire In Vitro, CHU de Tours, 37000 Tours, France; 5 Service de Neurochirurgie, CHU de Tours, 37000 Tours, France; 6 Service de Biochimie et Biologie Moléculaire, CHU d’Angers, 49000 Angers, France; pareynier@chu-angers.fr; 7 Mitovasc-Mitolab, UMR CNRS6015-INSERM1083, 49000 Angers, France; 8 Plateforme IBiSA de Microscopie Electronique, Université de Tours et CHU de Tours, 37000 Tours, France; sebastien.eymieux@univ-tours.fr (S.E.); emmanuelle.blanchard@univ-tours.fr (E.B.); 9 INSERM U1259, Université de Tours, 37000 Tours, France; 10 Service de Chirurgie Orthopédique et Traumatique, CHU de Tours, 37000 Tours, France; s.bakkouche@chu-tours.fr; 11 Service de Neurologie, CHU de Tours, 37000 Tours, France; luc.favard@univ-tours.fr

**Keywords:** Amyotrophic lateral sclerosis, metabolomics, mitochondria dysfunction, muscle, transcriptomics

## Abstract

Diverse biomarkers and pathological alterations have been found in muscle of patients with Amyotrophic lateral sclerosis (ALS), but the relation between such alterations and dysfunction in energetic metabolism remains to be investigated. We established the metabolome of muscle and serum of ALS patients and correlated these findings with the clinical status and pathological alterations observed in the muscle. We obtained data from 20 controls and 17 ALS patients (disease duration: 9.4 ± 6.8 months). Multivariate metabolomics analysis identified a distinct serum metabolome for ALS compared to controls (p-CV-ANOVA < 0.035) and revealed an excellent discriminant profile for muscle metabolome (p-CV-ANOVA < 0.0012). Citramalate was discriminant for both muscle and serum. High lauroylcarnitine levels in muscle were associated with low Forced Vital Capacity. Transcriptomics analysis of key antioxidant enzymes showed an upregulation of *SOD3* (*p* = 0.0017) and *GLRX2(1)* (*p* = 0.0022) in ALS muscle. Analysis of mitochondrial enzymatic activity in muscle revealed higher complex II/CS (*p* = 0.04) and lower LDH (*p* = 0.03) activity in ALS than in controls. Our study showed, for the first time, a global dysfunction in the muscle of early-stage ALS patients. Furthermore, we identified novel metabolites to be employed as biomarkers for diagnosis and prognosis of ALS patients.

## 1. Introduction

Amyotrophic lateral sclerosis (ALS) is a fatal adult-onset neuromuscular disease characterized by selective degeneration of motor neurons, progressive wasting and paralysis of voluntary muscles. Due to clinical heterogeneity and absence of biological tools to diagnose ALS, the diagnosis delay averages 9-13 months [1]. Several pathophysiological processes, such as mitochondrial dysfunction, glutamate-mediated excitotoxicity and aggregation of misfolded proteins, contribute to cell death, but the triggering factor, the timing and the interaction of different cellular events remain unclear [2]. Important metabolism alterations described in ALS patients, such as hypermetabolism, glucose intolerance and a putative protective effect of lipids, have supported research on metabolism in patients, animal and cell models [3,4,5]. Diverse studies have advocated that muscle per se may be involved in ALS pathogenesis, especially due to its central role in energetic metabolism [6]. The link between muscular atrophy, muscle denervation, glucose intolerance and lipid metabolism is not established yet in ALS [3,7,8,9,10,11,12]; however, muscle inactivity, the type of muscular fiber or muscle mitochondrial dysfunction may play a key role in ALS pathology [13,14,15,16,17,18].

In the context of a long diagnosis delay and successive therapeutics failures, we explored the pathophysiological mechanisms involved in muscle loss in ALS to identify metabolic biomarkers. We investigated peripheral and muscle metabolism, via metabolomics strategies, and we established a link with oxidative stress, mitochondrial function and muscle structure. We compared ALS patients and controls, and we also analyzed subgroups of ALS patients, to assess the relationship between biological data and clinical characteristics.

## 2. Material and Methods

### 2.1. Subjects’ Recruitment

Patients were recruited in the protocol METABOMU (ClinicalTrials.gov Identifier: NCT02670226, N°IdRCB: 215-AO1629-40) from March 2016 to September 2020. All patients were informed about the data obtained and their right to access these data, according to articles L.1121-1 and R1121-2 of the French Public Health Code. Participants were aged between 18 and 75 years and were affiliated to the social security scheme. All participants gave informed consent and the Ethics Committee in human research approved the study (CPP:2016-R3). This study was performed in line with the principles of the Declaration of Helsinki.

Patients were diagnosed for ALS according to the El Escorial criteria, and controls had no neurological disease. The exclusion criteria for both groups were: pregnant or breastfeeding women, contraindication to biopsy, contraindication to local anesthesia, treatment with oral or injectable anticoagulants, antiplatelet (except aspirin), unbalanced diabetes, systemic corticosteroid treatment, treatment against cramps or twitching that may affect muscle metabolism. Information on gender, age and Body Mass Index (BMI) were obtained for each subject included, in addition to site-of-onset (bulbar or limb-onset), age at diagnosis and age-at-onset for each patient. Age-at-onset was defined as the time at which motor weakness was first noted by the patient. Diagnosis delay was defined from the time of the first symptoms and the time of the diagnosis assignment. We obtained parameters of disease progression, such as the revised ALS Functional Rating Scale (ASLFRS-r), Forced Vital Capacity (FVC) and BMI at the diagnosis and during follow-up. We calculated the variation in ALSFRS-r, FVC, and weight and we established subgroups of ALS patients according to the median of variation. Based on the rapid evolution of the ALS cohort, we chose to analyze disease progression parameters over nine months. Disease duration of ALS was defined as the time since the first symptoms to the death, tracheostomy, or database lock.

### 2.2. Samples Collection

Serum and muscles samples were obtained from patients with ALS at the time of diagnosis (*n* = 17) and from matched controls (*n* = 20). Blood samples (collected in BD Vacutainer™ SST™ II Advance tubes, Thermo. Fisher Scientific Inc. Waltham, MA, USA) were centrifuged at 3000× *g* for 10 min, and the serum fraction was collected and conserved at −80 °C.

A muscle biopsy was obtained after local anesthesia and a short incision into the shoulder (deltoid muscle). Muscle samples were collected during a planned shoulder surgery for controls. Three muscle fragments were placed immediately into liquid nitrogen for preservation at −80 °C until analysis, and one was immediately fixed for electron microscopy.

### 2.3. Metabolomics Analysis

The protocol of metabolites’ extraction and mass spectrometry analysis has been previously reported [19,20]. Briefly, serum metabolites were extracted from 20 µL of serum with 100 µL of methanol. Muscle metabolites were extracted from 10 mg lyophilized muscle tissue. After lyophilization at 20 mbar at −20 °C for 48 h and pulverization, samples were weighed for data normalization. Metabolites were extracted from samples using methanol/water (1:1 *v*/*v*). Two extractions were performed using 750 µL of solvent and 1.4 mL of the extract was collected [21]. Information on liquid chromatography, quality controls and metabolites classification are detailed on Supplementary File S1 [22,23,24].

### 2.4. RNA Extraction and RT-qPCR Analysis

Muscle biopsies were homogenized in Trizol^®^ and RNA was obtained from the aqueous phase after chloroform addition. RNA was purified from the aqueous phase with the Zymo kit for RNA purification, according to manufacturer’s instructions (Zymo Research).

RNA quality was checked using a 2100 Bioanalyzer (Agilent, Santa Clara, CA, USA). RNA was reverse-transcribed using the SuperScript VILO cDNA Synthesis kit (Life Technologies, Carlsbad, CA, USA). The quantification of transcripts (Ensembl nomenclature) was achieved by reverse transcription-quantitative polymerase chain reaction using the Universal Probe Library technology (https://lifescience.roche.com, accessed on 5 May 2021) on a LightCycler 480 (Roche, Rotkreuz, Switzerland). Assays were designed to quantify either only one or several transcript variants at the same time (x,y,z…). All targets were analyzed simultaneously in triplicate and average values were used to determine relative quantification (RQ) values by the 2-ΔΔCt method [25].

### 2.5. Mitochondrial Enzymatic Activities

All muscle fragments were weighed and homogenized in mannitol buffer with a glass-glass Potter on ice, and centrifuged at 650× *g* and 4 °C for 20 min. The supernatant was decanted and retained. The pellet was resuspended in mannitol buffer (10 volume) and subjected to the same procedure. Both supernatants were pooled and used for the assays. The protein concentration was measured with the BCA protein assay kit (Thermo Fisher Scientific, Waltham, MA, USA). The enzymatic activities of NADH ubiquinone reductase (complex I), succinate ubiquinone reductase (complex II), ubiquinol-cytochrome c reductase (complex III), cytochrome c oxidase (complex IV), lactate dehydrogenase (LDH) and citrate synthase (CS) were carried out on the skeletal muscle homogenates at 37 °C a UVmc^2^ spectrophotometer (SAFAS, Monte Carlo, Monaco), according to a standard protocol [26]. Results were normalized to the CS activity; this Krebs cycle enzyme activity reflecting the mitochondrial content.

### 2.6. Electron Microscopy

Small muscle tissues were immediately fixed by incubation in 4% paraformaldehyde and 1% glutaraldehyde (Sigma, St-Louis, MO, USA) in 0.1 M phosphate buffer (pH 7.3), limiting the occurrence of artefacts and cell stress. After 24 h, samples were washed in Sorensen’s phosphate-buffered saline, post fixed by incubation for 1 h with 2% osmium tetroxide (Electron Microscopy Sciences, Hatfield, PA, USA). They were dehydrated in a graded series of ethanol solutions. Samples were embedded in Epon resin (Sigma), which was allowed to polymerize for 48 h at 60 °C. From Epon-embedded specimens, semi-thin sections (thickness 600 nm) were cut with a Leica EM UC7 ultramicrotome (Vienna, Austria) before being stained with toluidine blue (Dipath, Martinengo, Italy) for observation under a light microscope. Then, ultrathin sections (100 nm thick) were cut with a Leica EM UC7 ultramicrotome (Vienna, Austria), stained successively with 2.5% uranyl acetate (Merck, Darmstadt, Germany) and 1% lead citrate and deposited on electron microscope grids for examination observed under a JEOL 1011 (Tokyo, Japan) transmission electron microscope. Electron micrographs from three controls and three ALS patients were recorded with Gatan CEMOS RIO camera (Pleasanton, CA, USA).

### 2.7. Statistical Analysis

Univariate and multivariate analyses were performed to analyze the different datasets using GraphPad Prisma, JMP or Metaboanalyst. Full description of diverse statistical analyses used in this study is given in Supplementary File S1.

### 2.8. Data Availability

Metabolomics datasets are available as online Supplementary Material. Raw data sets are available upon request to the Corresponding author.

## 3. Results

### 3.1. Cohort Description

Seventeen ALS patients and 20 controls were recruited and followed in the CHRU of Tours from March 2016 to December 2020 (database lock). Clinical data and parameters for disease progression are presented in Table 1. Comorbidities, other medications, and anesthesia taken for the muscle biopsy are fully described in Supplementary File S2. At database lock, six patients were still alive and 11 were dead or lost to follow-up. Two patients presented mutations in the *C9ORF72* gene, but their disease evolution did not differ from the analyzed cohort.

### 3.2. Metabolomics of Serum

The complete list of the 155 analyzed metabolites is found in Supplementary File S3.

#### 3.2.1. Diagnosis Biomarkers

**Figure 1:**
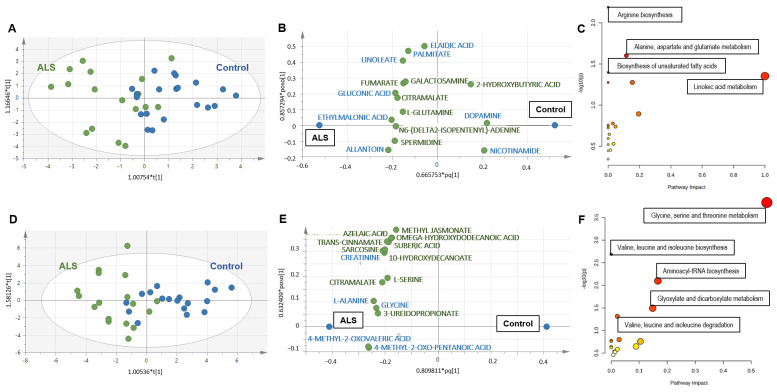
(**A**–**C**) Metabolomics analysis of serum from ALS and controls. (**A**) Score scatter plot based on the OPLS-DA models from serum to explain the diagnosis, with R2X = 0.452, R2Y = 0.477, Q2 = 0.269 and *p* < 0.035 for the CV-ANOVA test (blue: controls; green: ALS). (**B**) Loading scatter plot presenting the top 15 metabolites identified by the OPLS-DA. The horizontal axis displays the X-loadings p and the Y-loadings q of the predictive component. The vertical axis displays the X-loadings p(o) and the Y-loadings s(o) for the Y-orthogonal component. X-variables situated in the vicinity of the dummy Y-variables have the highest discriminatory power between the classes. The eight metabolites that had VIP scores higher than 1 are written in blue. (**C**) Pathway analysis with the 15 VIP metabolites highlighted alterations in arginine biosynthesis (*p* = 0.006), alanine, aspartate and glutamate metabolism (*p* = 0.02), biosynthesis of unsaturated fatty acids (*p* = 0.04) and linoleic acid metabolism (*p* = 0.04). Each node represents a metabolite set with its color based on its *p*-value and its size based on the pathway impact. The complete list of metabolic pathways is described in Supplementary File S4. (**D**–**F**) Metabolomics analysis of muscle from ALS and controls. (**A**) Univariate volcano plot analysis revealed different metabolites in the muscle metabolome from ALS patients and control subjects. Metabolites identified on the left are decreased in ALS patients compared to controls, while metabolites on the right of the diagram are increased in ALS patients. (**B**) Score scatter plot based on the OPLS-DA models from muscle to explain the diagnosis, with R2X = 0.76, R2Y = 0.555, Q2 = 0.446 and *p* < 0.0012 for the CV-ANOVA test (blue: controls; green: ALS). (**C**) Loading scatter plot presenting the top 15 metabolites identified by the OPLS-DA. The five metabolites that had VIP score higher than 1 are written in blue. (**D**) Pathway analysis with the 15 VIP metabolites highlighted alterations in the metabolism of glycine, serine and threonine (*p* < 0.001); biosynthesis (*p* = 0.002) and degradation (*p* = 0.04) of valine, leucine and isoleucine; aminoacyl-tRNA biosynthesis (*p* = 0.007) and glyoxylate and dicarboxylate metabolism (*p* = 0.03). The complete list of metabolic pathways is described in Supplementary File S4.

Univariate analysis of metabolites measured in serum samples revealed no difference between ALS and controls. Regarding multivariate analysis, unsupervised analysis (PCA) did not reveal any outsiders. OPLS-DA divided patients into ALS and control groups (Figure 1A) with correct performances defined by an R2X = 0.452, R2Y = 0.477 Q2 = 0.269, a significant CV-ANOVA test (*p* < 0.035) and a correct permutation test, ensuring the robustness of the model. The loading scatter plot shows the 15 most discriminant metabolites, with eight metabolites that had VIP scores higher than 1 (in blue; Figure 1B). The metabolic pathways associated with the 15 discriminant metabolites highlighted alterations in arginine biosynthesis, alanine, aspartate and glutamate metabolism, biosynthesis of unsaturated fatty acids (FAs) and linoleic acid metabolism (Figure 1C and Supplementary File S4).

#### 3.2.2. Biomarkers of Clinical Status

Within subgroups of ALS patients, volcano plot showed no discriminant metabolite between groups regarding clinical parameters at diagnosis. The multivariate models evaluating the relation between metabolites and clinical status were heterogeneous and the only significant associations with serum metabolome concerned ALSFRS-r (p-CV-ANOVA: 0.004, Supplementary Figure S1A,B) and age at onset (p-CV-ANOVA: 0.05; Supplementary Figure S1C,D).

#### 3.2.3. Prognosis Biomarkers

We excluded the FVC variation from the analysis because too many data were missing. Univariate analysis revealed several metabolites from serum that correlated with weight and ALSFRS-r variation: fifteen metabolites correlated with weight variation and two with ALSFRS-r variation (Table 2). However, no metabolite remained significant after correction for multiple tests.

PCA analysis did not reveal any outsiders. The PLS-DA model that divided ALS patients into groups according to disease progression showed that the model to explain weight variation was at the limit of significance (pCV-ANOVA = 0.07; Supplementary Figure S2A). Disease duration was associated with serum metabolome (pCV-ANOVA = 0.0008; Supplementary Figure S2D) and a correct permutation test. Discriminant metabolites associated with weight variation or disease progression, and pathway analysis are described in the Supplementary Material (Supplementary Figure S2 and Supplementary Files S5 and S6).

Cox proportional hazards modeling showed an association between survival and L-glutamic acid (*p* = 0.004) and L-tryptophan (*p* = 0.042), but only L-glutamic acid remained significantly associated with survival (*p* = 0.012) after multivariate analysis.

### 3.3. Metabolomics of Muscle

#### 3.3.1. Diagnosis Biomarkers

The analysis of the muscle metabolome obtained from ALS patients and controls revealed more differences than observed in serum. Volcano plot analysis revealed 15 discriminant metabolites, two decreased (C8-carnitine and lumichrome) and other 13 increased in ALS compared to controls (Table 2). The multivariate analysis also showed interesting findings. PCA did not reveal any outsiders and OPLS-DA divided patients into ALS and control groups (Figure 1D) with correct performances (R2X = 0.76, R2Y = 0.555, Q2 = 0.446, pCV-ANOVA < 0.0012) and a correct permutation test. The loading scatter plot shows the 15 most discriminant metabolites, with five metabolites that had VIP scores higher than 1 (in blue; Figure 1E). The metabolic pathways associated with the 15 discriminant metabolites highlighted a major impact in amino acids metabolism, aminoacyl-tRNA biosynthesis and the metabolism of glyoxylate and dicarboxylate (Figure 1F and Supplementary File S4).

#### 3.3.2. Biomarkers of Clinical Status

Within the subgroup of ALS patients, volcano plot showed no discriminant metabolite between groups determined from clinical parameters at diagnosis. The multivariate models showed heterogeneous, but significant, associations with site of onset (p CV-ANOVA: 0.017, Supplementary Figure S3A,B), FVC (p CV-ANOVA: 0.036, Supplementary Figure S3C,D), weight (p CV-ANOVA: 0.04, Supplementary Figure S3E,F) and age at onset (p CV-ANOVA = 0.004; Supplementary Figure S3G,H).

#### 3.3.3. Prognosis Biomarkers

Univariate analysis revealed that weight variation correlated only with one metabolite while ALSFRS-r variation correlated with the other three metabolites (Table 2). However, no metabolite remained significant after correction for multiple tests.

Multivariate (PCA) analysis of metabolomics, regarding clinical markers for ALS progression, revealed no outsiders. The PLS-DA model that divided ALS patients into groups, according to disease progression, showed that muscle metabolome was associated with weight variation (pCV-ANOVA = 0.04; Supplementary Figure S4A). Discriminant metabolites and pathway analysis are described in the Supplementary Material (Supplementary Figure S4B,C and Supplementary File S5). The model explaining disease duration from muscle metabolome was not significant (pCV-ANOVA = 0.12; Figure S3D,E; Supplementary File S6).

Survival analysis using Cox proportional hazards modeling revealed that muscle C10-carnitine (*p* = 0.047), C14-carnitine (*p* = 0.005), C8-carnitine (*p* = 0.020) and lauroylcarnitine (*p* = 0.003) were associated with survival (through univariate analysis), but multivariate analysis only highlighted C10-carnitine as significant (*p* = 0.047).

### 3.4. Common Metabolomics Alterations to Serum and Muscle

#### 3.4.1. Diagnosis Biomarkers

**Figure 2:**
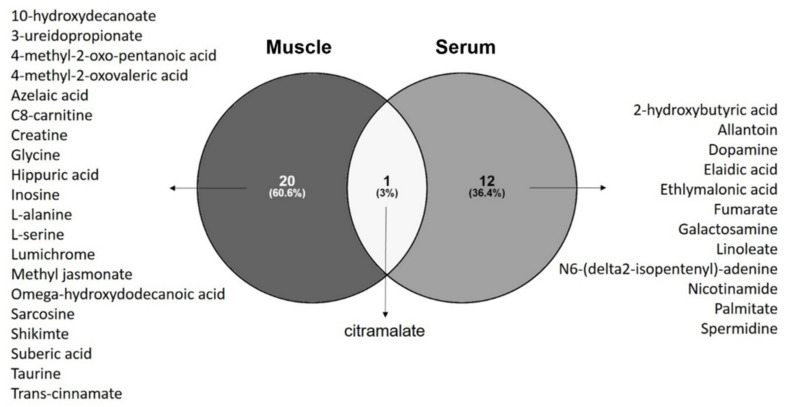
Venn diagram with discriminant metabolites revealed by univariate and univariate analysis identified 12 metabolites specific for serum of ALS patients, 20 metabolites specific to muscle of ALS patients, and citramalate as the metabolite commonly altered in serum and muscle of ALS patients when compared to control subjects. Venn diagram build with Venny 2.1.0 (https://bioinfogp.cnb.csic.es/tools/venny/index.html accessed on 15 April 2021).

By analyzing the discriminant metabolites of ALS patients in a Venn diagram, we observed that citramalate (a hydroxy-FA) is the only metabolite found in both serum and muscle that discriminates ALS patients from controls (Figure 2). Pathway analysis performed with discriminant metabolites from each tissue also revealed alterations in metabolic pathways that are common to the two matrices: beta-alanine metabolism; alanine, aspartate and glutamate metabolism; glutathione metabolism; and arginine and proline metabolism (Supplementary File S4).

#### 3.4.2. Biomarkers of Clinical Status and for Prognosis

Venn diagrams drawn from discriminant metabolites found in serum and muscle for each clinical parameter analyzed at diagnosis revealed that lauroylcarnitine was common to models explaining FVC and site at onset (Supplementary Figure S5A,B). Levels of muscle lauroylcarnitine, but not in serum, correlated negatively with FVC in both bulbar- and spinal-onset ALS (Supplementary Figure S5C,D).

Venn diagrams drawn from discriminant metabolites in serum showed that 5,6-dihydro-uracil was relevant in all models for disease progression; L-glutamic acid and tryptophan were common to models explaining disease duration and variation of weight; fumarate, linoleate and trans-aconitate were common to models explaining variation of ALSFRS-r and weight variation (Supplementary Figure S5E). No common metabolite for models based on muscle metabolome was found (Supplementary Figure S5F).

### 3.5. Antioxidant Genetic Profile of Muscle

**Figure 3:**
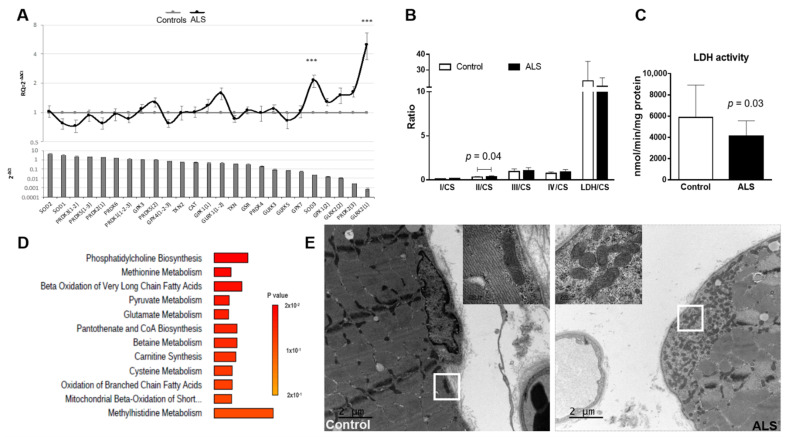
Alterations in muscle mitochondria. (**A**) Antioxidant genetic profile of ALS samples compared to controls. Expression levels of 25 transcripts of key antioxidant genes in the muscle of ALS patients (upper panel). Transcripts are ranked in decreasing order of expression in controls (bottom panel). Results are expressed as relative quantification (RQ) compared with control data (mean ± SEM). The horizontal gray line (y = 1) represents the healthy control profile (*n* = 20), and the black line represents patients’ data. Significant difference with the healthy controls (ΔCt values), *** *p* < 0.001. (**B**,**C**) Mitochondrial enzymatic activity from muscle of ALS patients and control subjects. (**B**) Ratios of analyzed complexes revealed alterations in the ratio Complex II/Cytrate synthase (II/CS; *p* = 0.04) and (**C**) in LDH activity (*p* = 0.03). No differences were found in the activity of the other complexes or ratios. Control: *n* = 20; ALS: *n* = 17. Results are shown as mean ± standard deviation. Data was analyzed using the Mann-Whitney statistical test. (**D**) Enrichment analysis of metabolites significantly correlated with LDH activity in muscle of ALS patients. Analysis performed with MetaboAnalyst tool. (**E**) Ultrastructural alterations in muscle mitochondria from ALS patients compared to control subjects. Representative transmission electron microscopy (TEM) images revealed the presence of mitochondria aggregates in the subsarcolemnic space in the muscle of ALS patient but not in the control subject. Scale bar: 2 µm. The insert corresponds to the zone indicated by the white box. Scale bar: 200 nm.

We performed an investigation of genes related to the antioxidant response in the muscle of ALS patients and controls. Antioxidant genetic profile showed a significant increase in the expression of *SOD3* (*p* = 0.0017) and *GLRX2(1)* (*p* = 0.0022) in ALS samples (Figure 3A).

We then correlated the levels of *SOD3* and *GLRX2(1)* expression with muscle metabolites. In controls, *SOD3* correlated negatively with levels of nine metabolites and positively with eight metabolites, mainly carnitine derivatives. In ALS patients, however, *SOD3* correlated negatively only with L-isoleucine and positively with four metabolites. *GLRX2(1)* expression in ALS patients, on the other hand, correlated only negatively with guanidinoacetate; while in control samples *GLRX2(1)* expression correlated with the levels of 23 metabolites. The names of significantly correlated metabolites, together with Spearman r and *p* values, are shown in Supplementary File S7.

### 3.6. Mitochondrial Enzymatic Activity in Muscle

Considering that an increase in oxidative stress is often associated with mitochondrial dysfunction, we analyzed the activity of mitochondrial enzymes in muscle of ALS patients and controls. Analysis of the mitochondrial enzymatic activity revealed higher complex II/CS in ALS than in controls (mean ± SD controls: 0.33 ± 0.06; ALS: 0.39 ± 0.09, *p* = 0.043; Figure 3B), and a lower lactate dehydrogenase activity in ALS muscle samples when compared to controls (mean ± SD controls: 5884 ± 3045 nmol/min/mg protein; ALS: 4097 ± 1458 nmol/min/mg protein, *p* = 0.033; Figure 3C). No differences were found when analyzing other complexes, nor in CS between groups (controls: 238.9 ± 57.9; ALS: 210.5 ± 48.3; *p* = 0.11); which reflects a similar mitochondrial mass in controls and ALS patients.

Interesting correlations were found regarding LDH activity and the levels of several metabolites. In muscle of ALS patients, LDH activity positively correlated with seventeen metabolites, mainly carnitine derivatives, while it correlated negatively with three metabolites. In the muscle of controls, LDH activity correlated positively with two metabolites. On the other hand, in ALS complex II/CS correlated negatively with the levels of four metabolites, while in controls the complex II/CS correlated positively with two metabolites. The complete list of significant metabolites (together with respective Spearman r and *p* values) is presented in Supplementary File S8. Enrichment analysis of metabolites significantly correlated with LDH activity in muscle showed that metabolites are especially involved in oxidation of FA and carnitine synthesis, confirming the mitochondrial dysfunction in ALS so far described (Figure 3D).

### 3.7. Electron Microscopy Analysis of Muscle

Since we found alterations in the enzymatic activity in muscle mitochondria from ALS patients, we decided to perform histological and microscopic analyses of samples from ALS and controls subjects to check for structural alterations in the muscle tissue and muscle mitochondria. Examination of semi-thin sections of muscle stained with toluidine blue of all patients revealed no major histological alterations when comparing ALS and controls. Even if there was a slight irregularity in the diameter and the contours of the muscle fibers, there was no grouping of the latter. There was no sign of obvious interfascicular fibrosis, inflammatory infiltrate, or even fat infiltrations.

Ultra-thin sections of three controls and three ALS patients examined by means of electron microscopy revealed mild aggregation of mitochondria in the subsarcolemnic compartment accompanied or not by glycogen accumulation in ALS samples. No abnormalities regarding the mitochondrial cristae or the presence of crystalline inclusions was noticed (Figure 3E).

## 4. Discussion

**Figure 4:**
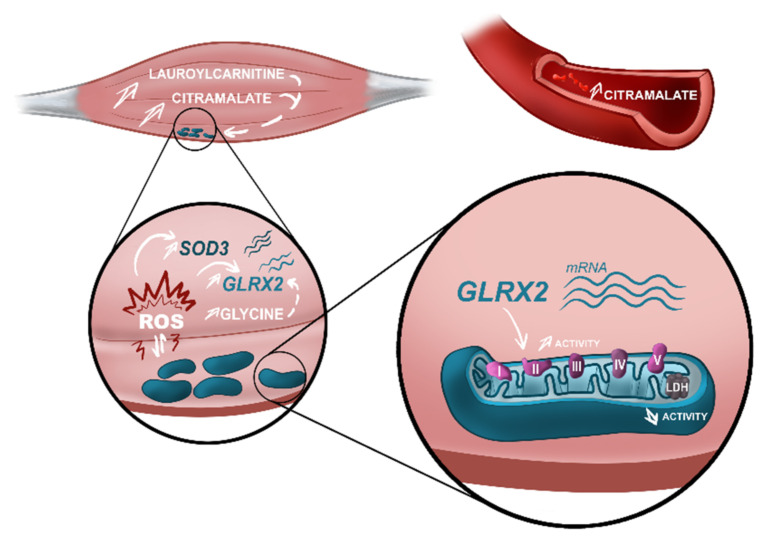
Alterations in actors of the energetic metabolism found in early-stage ALS patients. Metabolomics analysis performed in muscle and serum of ALS patients identified different metabolomes characterized by an increase in citramalate in both matrices. Besides this, increased levels of lauroylcarnitine were identified in the muscle of ALS patients as a bad prognostic factor. High levels of citramalate and lauroylcarnitine are associated with mitochondrial impairment. In ALS muscle, we observed a discrete accumulation of mitochondria in the subsarcolemnic space, suggestive of mitochondrial dysfunction. Mitochondrial dysfunction is a well-known source of reactive oxygen species (ROS). Transcriptomics analysis of muscle showed upregulation in ALS samples of two genes, SOD3 and GLRX2, that participate in the cellular antioxidant response. Furthermore, high levels of glycine—also found in the muscle of ALS patients—are associated with an upregulation of GLRX2. Upregulation of GLRX2 was shown to increase the activity of mitochondrial complex II, demonstrated in our analysis of mitochondrial activity in ALS muscle. Finally, mitochondrial dysfunction was also demonstrated by a decreased activity of LDH in ALS, compared to control samples. Our study confirmed the imbalance in muscle energetic metabolism in early-stage ALS and highlights metabolomics alterations associated with known pathological mechanisms described in ALS. These metabolomics alterations should be included in a panel of biomarkers to improve diagnosis and prognosis of ALS patients. Figure designed by Lucie Clarysse (Com&Sci).

In our study focusing on patients at diagnosis, we identified distinct serum metabolome profiles for ALS and controls, and we reported on, for the first-time, major alterations in the metabolome of muscles from ALS patients, compared to control subjects. Most of the metabolites identified as discriminant by the metabolomics models are related to known pathological mechanisms associated to ALS, which reinforces the notion that metabolomics analysis directly reflects the pathological alterations taking place in a tissue or an individual [27,28]. Accordingly, these findings were supported by targeted muscle transcriptomics and mitochondrial enzymatic activity (Figure 4), highlighting the early involvement of the energetic metabolism in ALS pathology.

### 4.1. The Validity of a Panel for Metabolic Biomarkers

#### 4.1.1. High Interest for Diagnosis Biomarkers

As the different techniques and types of analysis performed in prior “omics” studies preclude determining one single molecule being identified as biomarker, researchers in this field advocate for the advantage of providing a large metabolic panel, instead of single markers, to improve diagnosis and to identify commonly altered pathways [27,29,30].

In our metabolomics analyses, univariate analysis highlighted fifteen metabolites in the muscle (two being decreased and thirteen increased in ALS muscle, compared to control samples) that discriminate ALS from controls. Furthermore, multivariate analysis significantly distinguished ALS patients from controls in muscle and serum, with excellent performances in muscle. Different profiles of metabolites were also described by other groups in the serum [29,30] and in the plasma [31,32,33] of ALS patients compared to healthy subjects, which supports the hypothesis of different metabotypes [27]. A recent study also highlighted different pathways altered in the serum of ALS patients, including alanine, aspartate and glutamate metabolism, confirming our results [34]. However, no study has so far correlated both serum metabotype with metabotypes from tissues directly impacted by ALS pathophysiology. Importantly, as muscle is rarely explored in ALS, this is the first description of a specific metabotype for the muscle of ALS patients. The good performances achieved by our models reinforce the need for more investigations in larger cohorts. Application of such metabotypes to diagnose, or to establish the prognosis, of ALS patients would be more reflective of a condition known to be very heterogeneous in its clinical presentations [35,36]. 

Our findings highlight disturbance in the energetic metabolism in ALS patients, as shown by the main pathways identified: (1) alterations in the metabolism of unsaturated FA described in the serum, combined with (2) alterations in carbohydrates metabolism described in the muscle via the metabolism of glyoxylate and dicarboxylate (since the glyoxylate cycle allows humans to use fats for the synthesis of carbohydrates [37], and changes in carbohydrate metabolism were already reported in ALS patients [3,38,39]); and (3) alteration of amino acid pathways (serum: arginine biosynthesis, and alanine, aspartate and glutamate metabolism, muscle: metabolism of glycine, serine and threonine; biosynthesis and degradation of valine, leucine and isoleucine). Furthermore, our results support the search for biomarkers in different matrices to improve ALS diagnosis. Considering the multifactorial pathological mechanisms involved in ALS, and the different metabolic pathways associated with such mechanisms, it is necessary to search and validate a panel of biomarkers from different sources through different techniques to improve the diagnosis of patients. Even if our findings originated from a small cohort, we here reported relevant information about early pathophysiological mechanisms of ALS, laying ground for further research to assess and validate the interest of these muscle candidates in larger cohorts of ALS patients.

#### 4.1.2. Mild Interest for Prognostic Biomarkers

Most of the prognosis models performed in our study were significant but the lack of study power, inherent to the size of the cohort, limits the scope of our discussion. Of note, our model for serum metabolome explained disease duration, similar to a previous report showing the interest of a serum metabolomics profile to follow disease progression in ALS patients [29]. Regarding muscle metabolome, our model was significant in explaining weight variation in ALS patients. Interestingly, most pathways altered in the muscle of ALS patients were associated with amino acid metabolism and energetic metabolism; which is consistent with the hypermetabolism of ALS patients associated with decreased fat free mass [40]. Our metabolomics models also showed that disease duration was associated with levels of L-glutamic acid, confirming the key role of glutamate in ALS as reported by diverse studies [27,41].We also found that high levels of lauroylcarnitine in muscle of ALS patients were associated with low FVC. Lauroylcarnitine is also known as a pro-inflammatory saturated FA and is associated with metabolic-syndrome, adipose inflammation and glucose intolerance [38,39]. Glucose intolerance was reported in ALS patients [12,42,43,44], along with several alterations in glucose metabolism in both ALS patients and models [3,45,46,47]. Several studies have reported that glucose intolerance is associated with low pulmonary function [48,49,50], and a study performed in ALS patients submitted to tracheostomy and invasive ventilation found an increase in the incidence of hyperglycemia [51]. As low FVC is associated with a bad prognosis for ALS patients [52,53,54], lauroylcarnitine levels could be used as a combined biomarker for disease prognosis. We also found a correlation between lauroylcarnitine levels and survival, reinforcing the possible role of lauroylcarnitine as a biomarker for ALS prognosis. These promising results support the necessity to evaluate these correlations in a larger cohort with fast and slow progressions, through a longitudinal follow up study.

### 4.2. The Discriminant Ability of Citramalate in Muscle and Serum—New Biomarker for Diagnosis?

Here, we reported, for the first time, increased levels of citramalate in both serum and muscle of ALS patients. Citramalate, a metabolite once known as being exclusively present in yeast or anaerobic bacteria, is commonly used to diagnose gut dysbiosis in humans [55]. Alterations in gut microbiota can impact the gut-to-brain axis and have been associated with the occurrence of neurodegenerative diseases, including ALS [56,57,58]. Different microbiomes in ALS mouse models and patients were reported, with consequent differences in microbiome-related metabolites [59,60,61]. Blacher et al. [60] reported that some gut commensals could improve ALS symptoms while others would worsen these symptoms. Considering that the metabolites produced by such bacteria correspond to the systemic link between gut dysbiosis and motor neuron death in the CNS, they showed, by untargeted metabolomics, that beneficial bacteria induced the production of high levels of nicotinamide, and that nicotinamide supplementation improved motor function in an ALS mouse model. They also showed that nicotinamide levels are decreased in the serum and in the cerebrospinal fluid of ALS patients when compared to healthy controls [60]. Interestingly, we also found decreased levels of nicotinamide in the serum of our cohort of ALS patients—a metabolic alteration that is possibly related to the gut microbiome of these patients, as suggested by the increased levels of citramalate.

Citramalate was not so long ago described as a human metabolite [62]. Citramalate is analogous to malate, an important metabolite for the malate-aspartate shuttle in mitochondria. Besides providing electrons for oxidative phosphorylation, the malate-aspartate-NADH shuttle also generates glutamate and aspartate, and we showed impaired alanine, aspartate and glutamate metabolism in the serum of ALS patients. Dysfunctions in the malate-aspartate shuttle increase the vulnerability of neurons to glycolysis impairment and were demonstrated in cellular ALS models [63,64,65]. Further studies should focus on understanding the molecular mechanisms involved with the possible toxic effect of high citramalate levels, and to validate the application of this new putative biomarker for ALS diagnosis.

### 4.3. Consistency between Metabolomics Alterations and Pathophysiological Findings

#### 4.3.1. Oxidative Stress Revealed by Superoxide Dismutase 3 (SOD3) and Glutaredoxin-2 (GLRX2(2)) Deregulation Combined with Amino Acids Dysmetabolism

The increase in reactive oxygen species (ROS) is a well-known pathological player in ALS. Pathway analysis of discriminant metabolites in muscle revealed major alterations in pathways linked to the metabolism and biosynthesis of several amino acids and in aminoacyl-tRNA biosynthesis, and it was shown that increase in ROS impairs the correct activity of aminoacyl-tRNA, thus increasing the mistranslation of proteins and their incorrect folding [66]. Interestingly, we found increased mRNA levels of superoxide dismutase 3 (*SOD3*) and glutaredoxin-2 (*GLRX2(2)*), two proteins that participate in the cellular response to ROS. Increase in the expression of SOD3 mRNA was shown in mature C2C12 myotubes submitted to oxidative stress [67], and multi “omics” data analysis suggested SOD3 as a target for therapeutic purposes in AD [68]. The glutaredoxins system plays a key role in the pathophysiology of neurodegenerative diseases, such as Friedreich’s ataxia, Parkinson’s disease and AD [69,70,71]. Regarding glutaredoxin-2, studies performed on cells showed that the glutaredoxin system could decrease mutant SOD1 aggregates [72,73]. The overexpression of glutaredoxin-2 increases the solubility of mutant SOD1 in mitochondria, interferes with mitochondrial fragmentation by modifying the expression pattern of proteins involved in mitochondrial dynamics, preserves mitochondrial function and strongly protects neuronal cells from apoptosis [74]. Furthermore, glutaredoxin-2 is part of a highly regulated antioxidant system that maintains redox status in muscle mitochondria [75]. It was shown that glutaredoxin-2 induces S-glutathionylation of mitochondrial complex II in skeletal muscle [76] and that this post-translational modification can increase complex II activity [77], corroborating the results obtained here (i.e., increased activity of the mitochondrial complex II). These studies confirm the role of these antioxidant proteins in the cellular response to oxidative stress.

We observed that glycine was increased in muscle of ALS patients, compared to controls. Interestingly, glycine is increased in skeletal muscle in response to mitochondrial dysfunction [78]; an increase that, in turn, leads to the activation of the glutathione pathway as a defense mechanism against oxidative stress [79,80,81]. Furthermore, glycine was shown to induce protection against oxidative stress through increased expression of two important antioxidant proteins, Nrf-2 and HO-1 [82]. Increased glycine levels, together with increased expression in the mRNA of antioxidant proteins observed in ALS patients suggests an attempt of muscle cells to cope with the increase in ROS production, possibly due to impaired mitochondrial activity.

#### 4.3.2. Alterations in Mitochondrial Function and Distribution Reflect in the Intermediary Energetic Metabolism

Mitochondrial dysfunction associated with metabolism deregulation is a known pathological actor in ALS [83,84,85,86,87]. As confirmed by the present study, alterations in levels of several amino acids in samples from ALS patients highlighted a change in amino acid metabolism. We found that both biosynthesis and degradation of branched amino acids (BCAAs; valine, leucine and isoleucine) are disturbed in the muscle of ALS patients. In muscle, BCAAs not only provide a nonspecific carbon source of oxidation for production of energy but also act as a precursor for muscle protein synthesis [88]. This led to the hypothesis that BCAA supplementation could be used as a therapy for ALS, but trials studying the effect of BCAA treatment on ALS patients did not show any improvement [89,90,91]. As BCAAs are an important source of energy and promote glucose metabolism and glycogen synthesis [92], our finding of an impaired metabolism of BCAA further strengthens the hypothesis of a deficit in the energetic metabolism in ALS muscle.

The protective role of lipids in ALS patients is controversial [93,94], and research performed in animal models of ALS found an increased clearance in peripheral lipids [95] associated with a metabolic switch from glycolysis to lipid metabolism in early stages, which can, in the long term, induce oxidative stress [96]. We found that the biosynthesis of unsaturated FA was impaired in ALS serum. Similar alterations in plasma of ALS patients were reported by a previous metabolomics study [33]. Unsaturated FAs constitute another important source of energy after beta-oxidation in the mitochondria [97,98]. Since mitochondrial function is impaired in ALS, it is expected that one should observe a dysfunction in their metabolism. As FAs have other important cellular roles beyond energetic metabolism, as mediators of gene expression [99] and cell response to oxidative stress [100,101,102], more studies should be performed to understand the broad pathological impact of the impaired metabolism of unsaturated FAs in ALS.

Although a previous study did not find any difference in mitochondrial respiratory chain function [103], other studies confirmed an impaired mitochondrial function in the muscle of ALS patients [13,18,104,105,106] and more recently in hiPSC-derived myotubes [107,108]. Our analysis of ALS muscle revealed an increase in complex II activity and a decrease in LDH activity. Muscle LDH activity was decreased in the wobbler mouse, a murine model in which denervation followed degeneration of spinal cord motor neurons [109]. Our analysis also showed that LDH activity correlated positively with the levels of several carnitine derivatives and with lactate, and negatively with levels of α-glucose-1-phosphate in ALS muscle samples, reflecting an attempt of compensation for the energetic deficiency caused by mitochondrial impairment [110,111].

Ultrastructural analysis of muscle mitochondria revealed discrete alterations. As reported by previous studies, we found an accumulation of mitochondria in the subsarcolemnic space in muscle from ALS patients, while a normal distribution of mitochondria was observed in muscle from controls [112,113,114]. However, our analysis did not reveal the presence of giant mitochondria, nor para-crystalline inclusions or abnormal cristae, as reported before [114]. Ultrastructural alterations in muscle from two different animal models of ALS were reported: in *Drosophila melanogaster* expressing human SOD1 [115] and in the SOD1-G93A mouse [116]. While in these two models the observed alterations were probably due to the impaired activity of SOD1 [117,118,119], no patient carried SOD1 mutations in our cohort (and no description was found in the publication of Chung and Suh [114]), suggesting that these alterations are independent from the genotype. Ultrastructural alterations in mitochondria were already reported for mitochondria in the spinal cord [120] and more recently in fibroblasts from patients with and without pathogenic mutations [121], confirming the systemic impairment in mitochondrial function that underlies ALS pathology.

## 5. Conclusions

This study is the first one to perform a broad exploration of muscle in ALS patients, including metabolomics, exploration of mitochondrial structure and function as well as oxidative stress, in combination with serum metabolome profile. Even if performed in a small cohort, our investigation in muscle and serum, by a combination of complementary techniques, allowed us to highlight metabolic disturbances that were associated with increase in the response to oxidative stress and impairment in mitochondrial function in the muscle of ALS patients. Our findings strengthen the theory of key muscle dysfunction in the pathology of ALS and describe new metabolic actors for ALS disease. The combination of metabolomics analysis and more targeted pathological investigations opens the perspective of multiparametric explorations through innovative technologies and helps to shed light on pathophysiological mechanisms, novel biomarkers and new targets for efficient therapy in ALS.

## Figures and Tables

**Table 1:** Clinical characteristics of ALS patients and control subjects.

Data	Controls	ALS	*p*
Age	56.9 ± 19.2	65.9 ± 9.9	0.0793
Sex (men)	8/20 (40%)	8/17 (47%)	0.746 *
Weight (kg)	69.4 ± 13.6	64.2 ± 15.8	0.299
BMI (kg/m^2^)	24.9 ± 3.8	23.5 ± 4.0	0.3024
Age at onset (years)	-	65.0 ± 9.5	
Disease duration (from onset; months)	-	11.1 ± 6.8	
Diagnosis delay (from onset; months)	-	9.3 ± 4.7	
Spinal onset	-	64.7%	
FVC at diagnosis (%)	-	98.0 ± 7.1	
ALSFRS-r at diagnosis		34.7 ± 7.2	
*Parameters of disease progression*			
FVC variation (%)		−19.66 ± 10.6	
Weight variation (%)		−4.65 ± 9.0	
ALSFRS-r variation (%)		−23.43 ± 17.9	

Results are presented as means ± SD. Data was analyzed using unpaired *t* test (with Welch’s correction when necessary) or Fisher’s exact test for sex (*).

**Table 2:** Discriminant metabolites associated with clinical parameters of diagnosis or prognosis in serum and muscle.

**Muscle**			
**Metabolites**	***p* Value**	**Metabolites**	***p* Value**
***Diagnosis***		***Prognosis—ALSFRS-r***	
C8-carnitine	0.075	4-guanidinobutanoate	0.0202
Lumichrome	0.09	Glucuronolactone	0.0299
L-alanine	0.007	Sn-glycero-3-phosphocholine	0.0482
3-ureidopropionate	0.009		
Glycine	0.009	***Prognosis—weight variation***	
Citramalate	0.04	4-hydroxy-L-proline	0.0105
4-methyl-2-oxo-pentanoic acid	0.04		
Taurine	0.05		
4-methyl-2-oxovaleric acid	0.05		
Hippuric acid	0.06		
10-hydroxydecanoate	0.06		
Shikimate	0.07		
Inosine	0.07		
Suberic acid	0.09		
Trans-cinnamate	0.09		
**Serum**			
**Metabolites**	***p* Value**	**Metabolites**	***p* Value**
***Prognosis—weight variation***		***Prognosis—ALSFRS-r***	
5,6-dihydro-uracil	0.0023	10-hydroxydecanoate	0.043
Deoxycarnitine	0.0032	Elaidic acid	0.0456
Trans-aconitate	0.0057		
L-glutamic acid	0.0071		
Linoleate	0.0116		
L-tryptophan	0.013		
Malate	0.0159		
Isocitric acid	0.0192		
Succinate	0.0258		
O-acetyl-carnitine	0.0273		
Adipic acid	0.0406		
Nicotinate	0.0414		
Lactate	0.0448		
Cytidine	0.0459		
Norleucine	0.0466		

## Data Availability

Metabolomics datasets are available as online Supplementary Material. Raw data sets are available upon request to the Corresponding author.

## Supplementary Materials

The following supporting information can be downloaded at: https://www.mdpi.com/article/10.3390/biomedicines10061307/s1, Supplementary File S1: Supplementary methods; Supplementary File S2: Characteristics of ALS patients and controls included in this study; Supplementary File S3: List of metabolites analyzed by LC-MSMS from serum and muscle of ALS patients and controls; Supplementary File S4: Pathway analysis based on the metabolites identified in the muscle and the serum of ALS patients and controls; Supplementary File S5: Pathway analysis based on the metabolites identified in the serum and the muscle of ALS patients regarding weight variation; Supplementary File S6: Pathway analysis based on the metabolites identified in the serum and in the muscle of ALS patients regarding disease duration; Supplementary File S7: Correlation between muscle metabolites with levels of *SOD3* and *GLRX2(1)* expression from ALS patients and controls; Supplementary File S8: Correlation between metabolites from muscle or serum with LDH activity or the ratio complex II/CS from ALS patients and controls; Supplementary Figure S1: Metabolomics findings from serum and clinical parameters of ALS at diagnosis; Supplementary Figure S2: Multivariate analysis of the metabolome profile from serum of ALS patients and the variation of weight and disease duration. Supplementary Figure S3: Metabolomics findings in muscle and clinical parameters of ALS at diagnosis. Supplementary Figure S4: Multivariate analysis of the metabolome profile muscle of ALS patients and the variation of weight and disease duration. Supplementary Figure S5: Analysis of the most discriminant metabolites from both serum and muscle of ALS patients.
