# Supplementary material for: Metabolic Profile and Pathological Alterations in the Muscle of Patients with Early-Stage Amyotrophic Lateral Sclerosis
Source: Biomedicines. 2022 Jun 2;10(6):1307. doi: 10.3390/biomedicines10061307 (PMC9220134; doi:10.3390/biomedicines10061307)
Supplement: Supplementary file 1 [file biomedicines-10-01307-s001.zip › biomedicines-1713771-supplementary.pdf]

Metabolic profile and pathological alterations in the muscle of patients with early-stage amyotrophic lateral sclerosis.

Débora Lanznaster*, Clément Bruno*, Jérôme Bourgeais, Patrick Emond, Ilyess Zemmoura, Antoine Lefèvre, Pascal Reynier, Sébastien Eymieux, Emmanuelle Blanchard, Patrick Vourc'h, Christian R. Andres, Salah Bakkouche, Olivier Herault, Luc Favard, Philippe Corcia, Hélène Blasco. *equal author contribution

Corresponding author: Débora Lanznaster, PhD; Email: debora.lanznaster@univ-tours.fr

Supplementary File S1.

1. Metabolomics Analysis.

An UPLC Ultimate WPS-3000 system (Dionex, Germany) coupled to a Q-Exactive mass spectrometer (Thermo Fisher Scientific, Germany) and operated in positive (HESI+) and negative (HESI−) heated electrospray ionization modes (analysis for each ionization mode) was used. Liquid chromatography was performed using two different columns to increase the metabolic coverage. First, samples were injected on a Phenomenex Kinetex 1.7 µm XB – C18 column (150 mm x 2.10 mm) maintained at 55°C. The mobile phase gradient (preceded by an equilibrium time of 3 min) was maintained at a flow rate of 0.4 mL/min. The multistep gradient was programmed as follows: 0–2 min, 0.1% B; 2−6 min, 0.1−25% B; 6−10 min, 25−80% B; 10−12 min, 80−90% B; 12−16.5 min, 90-99.9% B; 16.5-20 min, 99.9–0.1% B. The second analyze was realized on a hydrophilic interaction liquid chromatography (HILIC) column (150 mm x 2.10 mm, 100 Å). The gradient was maintained at a flow rate of 0.3 mL/min. The multistep gradient was programmed as follows: 0−1.5 min, 95% B; 1.5−8 min, 95−82% B; 8-15 min, 82–75% B; 15-15.5 min, 75−25% B; 15.5−16 min, 25-3% B; 16-22 min, 3–95% B. The volume of injection was 5µL. During the full-scan acquisition, which ranged from 58 to 870 m/z, the instrument operated at 70,000 resolution (m/z = 200). Source parameters were maintained as described previously[1].

Pre-analytical and analytical steps of the experiment were validated by Quality Control (QC) samples (mix of 20 samples). Coefficients of variation (CV% = (the standard deviation/ mean) x 100), were calculated from all metabolites data. Metabolites with a CV in QCs >30% were excluded from the final dataset. QCs were analyzed at the beginning of the run, every 10 samples and at the end of the run.

A targeted analysis was applied on the samples, based on a library of standard compounds (Mass Spectroscopy Metabolite Library (MSML®) of standards, IROA Technologies™). The following criteria were used to identify the metabolites: (1) retention time of the detected metabolite within ± 20 s of the standard reference, (2) exact measured molecular mass of the metabolite within a range of 10 ppm around the known molecular mass of the reference compound, and (3) correspondence between isotopic ratios of the metabolite and the standard reference. The signal value was calculated using Xcalibur® software (Thermo Fisher Scientific, San Jose, CA) by integrating the chromatographic peak area corresponding to the selected metabolite.

2. Statistical analysis.

2.1 Univariate analysis

Comparison of continuous parameters defining population characteristics and mitochondrial enzymes data was done by Student or Wilcoxon test, depending on the distribution's normality (according to Shapiro test) and qualitative data were compared by the Chi^2 test. All data were checked for outliers by the Grubb's test. Correlation between metabolome data, mitochondrial enzymes and antioxidant genes were performed by the Spearman test, while the relation between FVC and lauroylcarnitine levels was evaluated by Pearson correlation (GraphPad Software version 7; GraphPad Software Inc.)

Metabolomics data was non-normalized, log-transformed and auto-scaled. The univariate analysis of metabolites levels between groups (ALS/controls and groups related to disease evolution) was based on fold-change (FC) values and the threshold of significance after non-parametric Wilcoxon test. This analysis was represented on a volcano plot using Metaboanalyst, version 5.0 (www.metaboanalyst.ca/). The x-axis represents the fold change between the subject groups (log scale). The y-axis represents the p-value for t-tests of differences between samples (negative log scale). False Discovery Rate (FDR) was used to account for multiple tests, thus highlighting only the most discriminant parameters. We highlighted only metabolites with $p<0.1$ and FC (ALS/Controls)>1.2 or <0.8. In case of comparison between more than two groups, Kruskal Wallis test was performed.

2.2 Metabolomics multivariate analysis

Multivariate analysis was performed to evaluate the relation between metabolome (in muscle and serum) and diagnosis as well as clinical evolution within the ALS cohort (based on the percentage of variation of BMI, ALSRFS-r and disease evolution). Data were Unit variance (UV)-scaled and log-transformed and were analyzed using SIMCA (v15).

Classification was performed by unsupervised Principal Component Analysis (PCA) to evaluate distribution of samples and identify outsiders. Supervised analysis based on Orthogonal Partial Least-Squares Discriminant Analysis (OPLS-DA) or PLS-DA (Partial Least-Squares Discriminant Analysis) enabled the evaluation of discrimination (determined by groups or values of quantitative parameters). OPLS-DA was cross-validated by withholding one-seventh of the samples in seven successive simulations such that each sample was omitted once in order to guard against overfitting,

leading to OPLS-DA built from one so called "predictive" component and two or more orthogonal components. The quality of the models was described by the cumulative modeled variation in the X matrix R2X(cum), the cumulative modeled variation in the Y matrix R2Y(cum), and the cross validated predictive ability Q2(cum) values. OPLS-DA was represented as a scatter plot of the scores t1 vs t1o. This plot represents the separation of the two classes of observations that occurs in the horizontal (t1) direction and the vertical (t1o) direction expresses within class variability. The default OPLS-DA loadings scatter plot displays the relation between the X-variables and the Y-variables for the first predictive component and the first Y-orthogonal component. Values of Variable Influence on Projection (VIP) represent the importance of the metabolites for the models. We performed a model optimization by variable exclusion to retain the 15 most discriminant metabolites. The most relevant variables were determined from the VIP and loading values scaled as correlation coefficients (pcorr).

The significance of the OPLS-DA model was evaluated by: 1) the Q2Ycum (goodness of prediction, >0.4 being a good predictive model), and the R2Ycum (goodness of fit) values. Q^2 is the fraction of the total variation of X or Y that can be predicted by a component, as estimated by cross-validation and R^2Y and R^2X display the fraction of the sum of squares for the selected component, 2) CV-ANOVA (ANalysis Of VAriance testing of Cross-Validated predictive residuals) test that is a diagnostic tool for assessing the reliability of the models, 3) permutation test, that is based on the comparison of the goodness of fit (R2 and Q2) of the original model with the goodness of fit of several models based on data where the order of the Y-observations has been randomly permuted, while the X-matrix has been kept intact. The permutation plot shows, for a selected Y-variable, on the vertical axis the values of R2 and Q2 for the original model (far to the right) and of the Y-permuted models further to the left. The horizontal axis shows the

correlation between the permuted Y-vectors and the original Y-vector for the selected Y. Considering the size of the cohort we did not validate metabolites/metabolome profile in an independent cohort to fulfill compulsory criteria of the use of biomarkers in routine practice, but we checked the reliability of the models to ensure robustness to pathway analysis.

2.3 Pathway Analysis

We constructed Venn diagrams to reveal the metabolites 1) in muscle and serum most significantly associated with the diagnosis or 2) associated with the four parameters chosen to evaluate disease evolution (http://bioinformatics.psb.ugent.be/webtools/Venn/). Enrichment and pathway analysis were systematically performed from all discriminant metabolites highlighted in the OPLS or PLS-DA models. Metabolic pathway enrichment analysis and pathway topology analysis were conducted using MetaboAnalyst platform [2], which computes a single *p* value for each metabolic pathway. Pathway topology analysis applies graph theory to measure a given experimentally identified metabolite's importance in a pre-defined metabolic pathway.

Pathway analysis calculated pathway impact that represents a combination of the centrality and pathway enrichment results; higher impact values represent the relative importance of the pathway, relative to all pathways included in the analysis. The pathway impact value was calculated as the sum of importance measures of the metabolites, normalized by the sum of importance measures of all metabolites in each pathway [3]. Major criteria are used to perform an informative analysis regarding the quality of pathway data. The KEGG pathway database (http://www.genome.jp/kegg/pathway.html) was used to explore the highlighted metabolic pathways. We highlighted pathways that present $p<0.05$ and 2 or more hits between discriminant metabolites.

2.4 Survival

We performed a survival analysis to evaluate the influence of biochemical and clinical parameters on disease duration using the Cox proportional hazard model. After a collinearity check, multivariate Cox proportional hazard models explored factors significantly associated with survival. P values <0.05 were considered significant. Analyses were performed using the JMP statistical software version. 7.0.2 (SAS Institute, Cary, NC, USA).

References

1. Martias C, Baroukh N, Mavel S, *et al*. Optimization of Sample Preparation for Metabolomics Exploration of Urine, Feces, Blood and Saliva in Humans Using Combined NMR and UHPLC-HRMS Platforms. *Molecules*. Jul 6 2021;26(14)doi:10.3390/molecules26144111
2. Xia J, Wishart DS. MetPA: a web-based metabolomics tool for pathway analysis and visualization. *Bioinformatics*. Sep 15 2010;26(18):2342-4. doi:10.1093/bioinformatics/btq418
3. Assenov Y, Ramirez F, Schelhorn SE, Lengauer T, Albrecht M. Computing topological parameters of biological networks. *Bioinformatics*. Jan 15 2008;24(2):282-4. doi:10.1093/bioinformatics/btm554

Metabolic profile and pathological alterations in the muscle of patients with early-stage amyotrophic lateral sclerosis.

Débora Lanznaster, Clément Bruno, Jérôme Bourgeais, Patrick Emond, Illyes Zemmoura, Antoine Lefèvre, Pascal Reynier, Sébastien Eymieux, Emmanuelle Blanchard, Patrick Vourc'h, Christian R. Andres, Salah Bakkouche, Olivier Herault, Luc Favard, Philippe Corcia, Hélène Blasco.
Corresponding author: Débora Lanznaster, UMR 1253, iBrain, Université de Tours, INSERM, Tours, France. Email : debora.lanznaster@univ-tours.fr.

Supplementary file S2. Characteristics of ALS patients and controls included in this study.

Identity	Group	Sex	Associated pathologies	treatments	Anesthesia	Anesthetics
C01	ALS	M	Arterial hypertension, dyslipidemia	Rilutek/Teglutik, Hexaquine, Forlax,Topalgic, Atropine, Hydrochlorothiazide, Amlodipine, Kardegic	Local	Lidocaïne
C02	ALS	W	Dysthyroidism	unknown	Local	Lidocaïne
C03	ALS	M	Dyslipidemia	unknown	Local	Lidocaïne
C04	ALS	W	Arterial hypertension, Thromboembolic pathology	Kardégic, Liorésal + HCT, Telmisartan, Ixprim	Local	Lidocaïne
C05	ALS	M	Depression	Rilutek, Tocopherol	Local	Lidocaïne
C06	ALS	W	Arterial hypertension, Asthma	Temerit, Tahor, Coaprovel, Seretide, Alprazolam, Rilutek, Tocopherol, Adrénaline, Laroxyl	Local	Lidocaïne
C07	ALS	W	unknown		Local	Lidocaïne
C08	ALS	M	None	Fluoxetine, Seroplex, Atropine collyre	Local	Lidocaïne
C09	ALS	W	None	Paracetamol, Tocopherol, Rilutek, Debridat	Local	Lidocaïne
C10	ALS	W	Epilepsy	Rilutek, Keppra, Laroxyl, Atropine collyre, Inexium, Tocopherol, Triflucan	Local	Lidocaïne
C11	ALS	M	Dyslipidemia	Rilutek, Topalgic, Transipeg, Atropine collyre	Local	Lidocaïne
C12	ALS	W	Arterial hypertension, Dyslipidemia	Aprazolam, Météospasmyl, Duphalac, Lercan, Kardegic, Inegy, Uvédose	Local	Lidocaïne
C13	ALS	W	Tumor pathology, Dyslipidemia	Topalgic	Local	Lidocaïne
C14	ALS	M	None	Tocopherol, Rilutek, Levothyrox, Lexomil, Okimus, Effexor, Tercian, Imovane	Local	Lidocaïne
C15	ALS	W	Coronary arthery disease		Local	Lidocaïne
C16	ALS	M	None		Local	Lidocaïne
C17	ALS	M	Arterial hypertension		Local	Lidocaïne
T01	Control	W	Hypothyroidism, arterial hypertension, Hypercholesterolemy	Amlodipine, Fenofibrate, Dosulepine, Pravastatine, Levothyrox, Esomeprazole, Zolpidem, Bromazepam, Hydroxyzine, Prégabaline, Ketoprofène, Paracétamol, Enoxaparine, Cafzoline, Morphine, Ondansetron, Polyethylène glycol, Tramadol, Vitamine C	Loco-regional + general	Sufentanil, propofol, ketamine, ephedrine, phenylephrine, nefopam, ropivacaine
T02	Control	W	None	Prégabaline, Alprazolam, PEG, Ondansetron, Ketoprofene, Esomeprazole, Enoxaparine, Morphine, Paracetamol, Tramadol, Vitamine C, Cefazoline	Loco-regional + general	Midazolam, Sufentanil, Propofol, Ketamine, Atracurium, Nefopam, Droperidol, Dexamethasone, Ropivacaine
T03	Control	M	Arterial hypertension, atrial fibrillation, ischemic cardiopathy, aortic bioprosthesis, hypercholesterolemy	Trinitrine, Acetylsalicylate, rosuvastatine, Bisoprolol Ramipril, Pregabaline, Morphine, Ondansetron, Esomeprazole, Acupan, Paracetamol, Tramadol, Vit C	Loco-regional + general	Sufentanil, Ketamine, Propofol, Atracurium, Dexamethasone, Ephedrine, Droperidol, Ropivacaine

T04	Control	M	Arterial hypertension, Diabetes (type 1)	Hydrochlorothiazide, Metformine, Ramipril, Cefazoline, Lyrica, Lantus, Zopiclone, Venofer, Macrogol, Ondansetron, Ketoprofene, Esomeprazole Morphine, Paracetamol, Tramadol	Loco-regional + general	Sufentanil, Propofol, Atracurium, Dexamethasone, Morphine, droperidol, ketamine, Ropivacaine
T05	Control	W	Ankylosing spondylitis, Hypothyroidism	Cefazoline, Tramadol, Lexomil, Loprazolam Etodolac, Fentanyl, Levothyroxine, Pregabaline Morphine, Macrogol, Ondansetron, Ketoprofene Esomeprazole, Paracetamol, Lamaline, VitC	Loco-regional + general	Midazolam, Sufentanil, Propofol, Atracurium, Dexamethasone, Ketamine, Neosynephrine, Ephedrine, Ropivacaine
T06	Control	W	Arterial hypertension, Crohn's disease, bronchopneumopathie chronique obstructive, Breast cancer, hip sepsis	Cefazoline, Lyrica, Ondansetron, Paracetamol Morphine, Imovane, Cymbalta, Lamaline, Dafalgan Tramadol, Diclofenac, Trandolapril, Bisoprolol, Lercanidipine, Temesta, Zolpidem, Omeprazole, Bromazepam, Enoxaparine, Vitamine C, Ketoprofene	Loco-regional + general	Sufentanyl, ketamine, propofol, Atracurium, Lidocaine, Dexamethasone, Droperidol, Morphine
T07	Control	W	Cholesterolemy, depression	Bromazepam, Seropam, Morphine, Hydroxyzine Paracetamol, Ketoprofene, Pregabaline, Esomeprazole Ondansetron, Lamaline, Vitamine C, Amoxicilline (25/07/16)	Loco-regional + general	Droperidol, Dexamethasone, Sufentanil, Ketamine, Propofol, Atracurium, Ephedrine, Dalacine, Ropivacaine
T08	Control	M	None	Pregabaline, Ketoprofene, Paracetamol, Tramadol Omeprazole, Vitamine C	Loco-regional + general	Sufentanyl, Propofol, Atracurium, Aphedrine, Atropine,Ropivacaine, Ketamine
T09	Control	M	None	Pregabaline, Ketoprofene, Paracetamol, Lamaline, Vitamine C, Cefazoline	Loco-regional + general	Sufentanyl, Hypnovel, Propofol, Atracurium, Dexamethasone, Ketamine, Morphine, Droperidol
T10	Control	M	None	Pregabaline, Ketoprofene, Ondansetron, Paracetamol Tramadol, Omeprazole, Vitamine C, Vancomycine Tazocilline, Cefazoline, Amoxicilline, Sufentanyl Popofol, Ephedrine, Ondansetron, Esomeprazole, Tramadol	Loco-regional + general	Hypnovel, Sufentanyl, Ketamine, Propofol, Atracurium, Ropivacaine, Cefazoline, Morphine, Tramadol
T11	Control	W	Hepatitis C (cured)	Exacyl, Morphine, Ketoprofene, Paracetamol Ondansetron, Pregabaline, Esomeprazole, Vitamine C	Loco-regional + general	Sufentanyl, Midazolam, Ketamine, Propofol, Atracurium, Dexamethasone, Ephedrine, Droperidol
T12	Control	M	Arterial hypertension, Pulmonary embolsim, Cholesterolemy, Diabetes (type 1)	Kardegic, Pravastatine, Diastabol, Bipreterax, Metformine , Lansoprazole, Glibenclamide, Allpurinol, Ranidipine Nebivolol, Levothyrox, Lyrica, Morphine, Enoxaparine, Insuline Lantus, Augmentin, Ipratrpium, Terbutaline Paracetamol, ketoprofene, Zophren, Tramadol, Vit C	Loco-regional + general	Hypnovel, Sufentanyl, Ketamine, Propofol, Exacyl, Salbutamol, Ropivacaine, Ephedrine, Atrovent, Bricanyl, Droperidol
T13	Control	M	None	Pregabaline, Paracetamol, Ketoprofene, Morphine Droperidol, Ondansetron, Esomeprazole, Tramadol, Vit C	Loco-regional + general	Hypnovel, Sufentanyl, Ketamine, Propofol, Atracurium, Dexamethasone, Ephedrine

T14	Control	W	Arterial hypertension, breast cancer	Amlodipine, Valsartan, Esidrex, Uradipil, Morphine, Ketoprofene, Paracetamol, Pregabaline, Ondansetron Tramadol, Esomeprazole, Fer	Loco-regional + general	Sufentanyl, Midazolam, Ketamine, Propofol, Atracurium, Dexamethasone, Cefazoline, Exacyl, Ropivacaine
T15	Control	W	Gibert's disease	Morphine, Droperidol, Ketoprofene, Esomeprazole Pregabaline, Vit C, Paracetamol, Tramadol	Loco-regional + general	Hypnovel, Ketamine, Propofol, Dexamethasone, Atracurium, Neosynephrine, Cefazoline, Ropivacaine, Sufentanyl
T16	Control	W	None	Paracetamol, ketoprofene, Cefazoline, Ondansetron Vit C, Tramadol, Pregabaline	Loco-regional + general	Exacyl, Dexomethasone, Neosynephrine, Sufentanyl, Hypnovel, Ketamine, Propofol, Atracurium, Ephedrine, Ropivacaine
T17	Control	W	Dyslipidemy	Rosuvastatine, Pregabaline, Paracetamol, Tramadol Vitamine C, Morphine, Droperidol, Ketoprofene, Ondansetron, Esomeprazole	Loco-regional + general	Hypnovel, Ketamine, Sufentanyl, Propofol, Atracurium, Exacyl, Dexamethasone, Ephedrine, Ropivacaine
T18	Control	M	Arterial hypertension	Bisoprolol, Omeprazole, Spiramycine, Metronidazole, Pregabaline, Alprazolam, Paracetamol, Cefazoline Ketoprofene, Hydrochlorothiazide, Morphine, Droperidol Ondansetron, Tramadol, Vitamine C	Loco-regional + general	Hypnovel, Ketamine, Ropivacaine, Sufentanyl, Propofol, Atracurium, Exacyl, Dexamethasone
T19	Control	W	Arterial hypertension, Rhythm disturbances, gastroesophageal reflux, arthritis	Atenolol, Apranax, Pentoprazole, Lyrica, Simvastatine Clobetazol (shampoing), Uvedose, Flecaine, Valsartan, Ketoprofene, Morphine, Droperidol, Paracetamol, OndansetronEnoxaparine, Tramadol, Vitamine C, Infiltration radioguidée	Loco-regional + general	Ropivacaine, Prostigmine, Atropine, Ephedrine, Sufentanil, Diprivan, Rocuronium, Ketamine, Midazolam, Phenylephrine
T20	Control	W	Arterial hypertension, depression	Fosinopril, Seroplex, Atarax, Oestradiol, Vancomycine Morphine, Ondansetron, Esomeprazole, Venofer, Ketoprofene, Paracetamol, Pregabaline, naproxene, Vitamine C, Tramadol	Loco-regional + general	Sufentanil, propofol, Ketamine, Atracurium, Dexamethasone, Phenylephrine, Naropeine, Droleptan

Metabolic profile and pathological alterations in the muscle of patients with early-stage amyotrophic lateral sclerosis.

Débora Lanznaster, Clément Bruno, Jérôme Bourgeais, Patrick Emond, Illyes Zemmoura, Antoine Lefèvre, Pascal Reynier, Sébastien Eymieux, Emmanuelle Blanchard, Patrick Vourc'h, Christian R. Andres, Salah Bakkouche, Olivier Herault, Luc Favard, Philippe Corcia, Hélène Blasco.
Corresponding author: Débora Lanznaster, UMR 1253, iBrain, Université de Tours, INSERM, Tours, France. Email : debora.lanznaster@univ-tours.fr.

Supplementary file S3. List of metabolites analyzed by LC-MSMS from serum and muscle of ALS patients and controls. Mean and standard deviation as well as raw p-values are provided.

Muscle samples					
	ALS		Control		
	mean	standard error	mean	standard error	raw p-value
10-HYDROXYDECANOATE	0,0002373	9,57E-05	0,0001558	3,37E-05	4,92E-05
1-AMINOCYCLOPROPANE-1-CARBOXYLATI	0,0003485	9,56E-05	0,0003095	4,09E-05	0,0001289
1-OLEOYL-RAC-GLYCEROL	1,97E-05	4,88E-06	1,707E-05	2,92E-06	0,0003645
2-DEOXY-GLUCOSE	0,0071521	0,00193817	0,005728	0,00145356	0,0017158
2-HYDROXYPYRIDINE	1,95E-05	7,82E-06	1,666E-05	4,72E-06	0,0017158
3-AMINO-4-HYDROXYBENZOIC ACID	7,08E-06	4,69E-06	7,62E-06	3,65E-06	0,0019192
3-DEHYDROSHIKIMATE	2,59E-05	1,80E-05	3,03E-05	1,99E-05	0,0023938
3-HYDROXYBUTANOIC ACID	0,0004373	0,0002467	0,0004502	0,00011871	0,0026694
3-SULFINO-L-ALANINE	0,0004349	0,00083938	0,0001348	8,71E-05	0,0033094
3-UREIDOPROPIONATE	1,643E-05	0,00001133	6,82E-06	6,09E-06	0,0050251
4-AMINOBUTANOATE	0,0002509	7,66E-05	0,0002417	5,46E-05	0,0055642
4-GUANIDINOBUTANOATE	3,25E-05	2,49E-05	2,68E-05	0,00001905	0,0055642
4-HYDROXY-L-PROLINE	0,0001849	7,59E-05	0,0001494	0,00006302	0,0080299
4-METHYL-2-OXO-PENTANOIC ACID	0,0022193	0,00147803	0,0009739	0,00083917	0,008281
4-METHYL-2-OXOVALERIC ACID	0,004383	0,00285358	0,0020611	0,00180899	0,008281
5-HYDROXYINDOLEACETATE	1,30E-05	7,99E-06	1,39E-05	7,55E-06	0,0110416
5-HYDROXYLYSINE	0,0002507	0,00010407	0,0002174	8,09E-05	0,0121288
5'-METHYLTHIOADENOSINE	1,93E-06	1,85E-06	1,23E-06	7,38E-07	0,0145914
5-OXO-L-PROLINE	0,0001684	5,71E-05	0,0001421	2,36E-05	0,0159806
ADENINE	1,177E-05	2,14E-05	8,74E-06	0,00001375	0,0159806
ADENOSINE	6,03E-06	5,45E-06	4,10E-06	2,40E-06	0,0159806
ADENOSINE 5'-MONOPHOSPHATE	3,20E-05	9,32E-05	1,46E-06	2,48E-06	0,0174847
ALLANTOIN	6,22E-06	4,25E-06	6,76E-06	0,00001275	0,0191115
ALPHA-AMINOADIPATE	7,011E-05	4,55E-05	4,42E-05	0,00002347	0,0191115
ALPHA-GLUCOSE	0,0022141	0,00063313	0,0019832	0,00048572	0,022766
ALPHA-GLUCOSE 1-PHOSPHATE	1,112E-05	9,15E-06	1,55E-05	1,50E-05	0,022766
AZELAIC ACID	0,0153696	0,00672659	0,0106358	0,00144963	0,022766
BETAINE	0,0068781	0,0028287	0,0062028	0,00198387	0,022766
C10-CARNITINE	4,71E-06	4,13E-06	1,02E-05	8,82E-06	0,027013
C14-CARNITINE	2,69E-05	1,53E-05	4,76E-05	3,29E-05	0,027013
C16-CARNITINE	5,67E-05	2,57E-05	8,06E-05	0,00004965	0,0319273
C18-CARNITINE	7,37E-06	4,53E-06	1,14E-05	7,27E-06	0,0346595
C2-CARNITINE	0,0039318	0,00170781	0,0041185	0,0013375	0,039891
C3-CARNITINE	0,0004475	0,00022925	0,0004382	0,00013784	0,0407264
C4-CARNITINE	7,98E-05	4,72E-05	0,0001099	8,35E-05	0,0440831
C5-CARNITINE	6,10E-05	5,37E-05	4,612E-05	3,13E-05	0,0440831
C8-CARNITINE	6,11E-06	4,40E-06	1,48E-05	1,23E-05	0,0476704
CARNOSINE	0,0053794	0,00233373	0,0046249	0,00167656	0,0555837
CHOLINE	1,24E-05	6,55E-06	1,05E-05	3,21E-06	0,0555837
CITRAMALATE	2,69E-05	2,69E-05	6,97E-06	5,85E-06	0,0621942
CITRULLINE	0,0003811	0,00027577	0,0003095	0,00018677	0,0645625
CREATINE	0,0501782	0,01414327	0,0382527	0,00811664	0,0645625
CREATININE	0,0145781	0,00460812	0,01504	0,00298713	0,0645625
Cyclic AMP	7,14E-06	2,66E-06	7,49E-06	0,0000038	0,0747059
CYTIDINE	4,46E-05	4,82E-05	5,322E-05	4,09E-05	0,080246
CYTOSINE	4,42E-05	4,26E-05	5,37E-05	3,34E-05	0,080246
DEOXYCARNITINE	0,0045976	0,00201981	0,0039689	0,00120071	0,0923266

DIETHANOLAMINE	9,69E-05	5,84E-05	5,97E-05	2,17E-05	0,0988927
EPINEPHRINE	0,0002012	0,00008257	0,0001461	3,94E-05	0,0988927
ETHANOLAMINE	3,93E-06	1,09E-06	3,55E-06	8,25E-07	0,1058263
ETHANOLAMINE PHOSPHATE	7,07E-06	3,51E-06	6,02E-06	3,52E-06	0,1208203
ETHYLMALONIC ACID	7,09E-06	4,87E-06	4,54E-06	1,54E-06	0,1374852
FORMYL-L-METHIONYL PEPTIDE	1,288E-05	8,42E-06	1,16E-05	2,17E-06	0,1374852
FUMARATE	0,0004625	0,00033108	0,0003603	0,00011744	0,1374852
GALACTARATE	1,19E-05	1,12E-05	1,44E-05	9,13E-06	0,1464415
GAMMA-LINOLENIC ACID	1,22E-05	4,82E-06	1,22E-05	5,74E-06	0,1558374
GLUCONIC ACID	0,0001674	7,51E-05	0,0001196	2,57E-05	0,1558374
GLUCURONIC ACID	1,26E-05	3,41E-06	1,25E-05	3,02E-06	0,1558374
GLUCURONOLACTONE	1,62E-07	1,15E-07	1,43E-07	8,83E-08	0,1656838
GLUTATHIONE	0,0008861	0,00045645	0,0011526	0,00034439	0,1656838
GLYCERALDEHYDE 3-PHOSPHATE DIETHYI	0,0030678	0,0018334	0,0025873	0,00083367	0,1759912
GLYCERATE	0,0007599	0,00059432	0,0004724	0,00023485	0,1759912
GLYCINE	0,0001267	3,96E-05	8,28E-05	2,28E-05	0,1867694
GLYCOLALDEHYDE DIMER	8,20E-06	2,78E-06	7,17E-06	2,17E-06	0,1867694
GUANIDINOACETATE	1,872E-05	0,00000736	2,017E-05	8,11E-06	0,1867694
GUANINE	7,791E-05	0,00003491	7,405E-05	0,00003409	0,1980274
GUANOSINE	2,45E-05	1,78E-05	2,22E-05	1,07E-05	0,1980274
GUANOSINE 5'-MONOPHOSPHATE	3,33E-05	1,29E-05	3,17E-05	2,37E-05	0,1980274
HIPPURATE	4,58E-06	1,77E-06	2,89E-06	1,98E-06	0,1980274
HISTAMINE	0,0001654	0,00011325	0,0001344	6,50E-05	0,2097741
HOMOSERINE	0,0003705	0,00013784	0,0002883	8,21E-05	0,214812
HYPOXANTHINE	0,0219341	0,00629716	0,017415	0,00371858	0,2220171
INOSINE	0,0019242	0,00058392	0,0014815	0,00025098	0,2220171
INOSINE 5'-MONOPHOSPHATE	0,0067994	0,00213629	0,006645	0,00156218	0,2255778
LACTATE	0,2542663	0,06991638	0,229564	0,01621965	0,2347637
L-ALANINE	0,002031	0,00052499	0,0014234	0,00022606	0,2347637
L-ANSERINE	5,06E-05	2,64E-05	4,25E-05	1,91E-05	0,2617914
L-ARABITOL	3,11E-05	2,48E-05	2,06E-05	5,54E-06	0,2617914
L-ARGININE	0,0009807	0,0003465	0,0008978	0,00037224	0,2617914
L-ASPARAGINE	0,0001027	3,98E-05	8,615E-05	1,87E-05	0,2760823
L-ASPARTATE	1,501E-05	2,88E-05	3,98E-05	4,98E-05	0,2760823
LAUROYLCARNITINE	1,50E-05	9,30E-06	2,99E-05	2,31E-05	0,2908963
L-CARNITINE	0,1587535	0,04466533	0,1290186	0,02301093	0,2908963
LEUCINE	0,0004732	0,00012694	0,0005227	0,00012958	0,2908963
L-GLUTAMIC ACID	0,0023692	0,0007236	0,0021081	0,00050886	0,3055574
L-GLUTAMINE	0,0146936	0,00437012	0,0116359	0,0022161	0,3062358
L-HISTIDINE	0,0019591	0,00077598	0,0016318	0,00046163	0,3062358
L-ISOLEUCINE	0,0001344	4,40E-05	0,0001525	4,61E-05	0,322102
L-KYNURENINE	1,508E-05	8,10E-06	2,54E-05	1,41E-05	0,322102
L-LYSINE	0,0004045	0,00017035	0,000369	0,00016839	0,3384953
L-METHIONINE	0,0001108	4,10E-05	0,0001105	5,01E-05	0,3384953
L-ORNITHINE	3,915E-05	1,60E-05	2,69E-05	1,34E-05	0,3384953
L-PHENYLALANINE	0,0005044	0,00018296	0,000517	0,00017197	0,3554146
L-PIPECOLIC ACID	0,0001492	7,81E-05	0,0001236	6,27E-05	0,3728579
L-PROLINE	0,0047555	0,00162714	0,0036908	0,00094889	0,3728579
L-SERINE	0,0003269	0,0001076	0,0002478	0,0000639	0,3728579
L-THREONINE	0,0006245	0,00017996	0,0005188	7,18E-05	0,3906891
L-TRYPTOPHAN	0,0002849	9,90E-05	0,000286	0,00010563	0,3908219
L-TYROSINE	0,0003369	9,83E-05	0,0003162	0,0001067	0,3908219
LUMICHROME	5,06E-06	6,85E-06	9,61E-06	4,69E-06	0,3908219
L-VALINE	0,000533	0,0001306	0,0004976	9,29E-05	0,3908219
LYXOSE	4,04E-05	0,00002028	4,40E-05	1,46E-05	0,4282919
MALATE	0,0041952	0,00218534	0,0036877	0,00115607	0,4282919
MANNITOL	0,0003071	9,41E-05	0,0002806	9,19E-05	0,4282919
MANNOSE	8,85E-05	7,86E-05	6,41E-05	0,00004995	0,4477849
METHYL JASMONATE	1,077E-05	4,33E-06	8,10E-06	1,53E-06	0,4477849
METHYLGUANIDINE	2,77E-05	2,06E-05	3,47E-05	1,98E-05	0,4677724

MYO-INOSITOL	5,245E-05	0,00002806	5,16E-05	2,11E-05	0,4677724
N(PAI)-METHYL-L-HISTIDINE	9,108E-05	4,28E-05	8,24E-05	4,04E-05	0,4677724
N6-(DELTA2-ISOPENTENYL)-ADENINE	2,04E-05	1,38E-05	2,05E-05	1,28E-05	0,4882447
N-ACETYL-L-ASPARTIC ACID	1,37E-05	8,14E-06	1,12E-05	6,11E-06	0,4882447
N-ACETYL-L-METHIONINE	0,0001115	5,16E-05	0,000102	6,71E-05	0,4882447
N-ACETYL-L-PHENYLALANINE	3,55E-05	2,30E-05	3,067E-05	1,44E-05	0,4882447
N-ACETYLNEURAMINATE	1,78E-05	7,49E-06	2,10E-05	8,49E-06	0,4882447
N,N,N-TRIMETHYL-LYSINE	9,76E-06	2,93E-06	9,85E-06	4,65E-06	0,4882447
NICOTINAMIDE	0,0156191	0,00470442	0,0162493	0,00343638	0,4882447
NICOTINATE	5,22E-05	1,43E-05	4,21E-05	6,50E-06	0,5091907
O-ACETYL-L-CARNITINE	0,0356887	0,02214523	0,0330133	0,01857346	0,5091907
OLEATE	4,27E-06	1,38E-06	4,98E-06	1,80E-06	0,5091907
OMEGA-HYDROXYDODECANOIC ACID	0,0003059	8,77E-05	0,0002432	3,87E-05	0,5331531
OPHTHALMIC ACID	2,81E-05	1,43E-05	3,04E-05	0,0000138	0,541187
PALMITATE	1,85E-05	9,19E-06	1,61E-05	1,07E-05	0,5524529
PANTOTHENIC ACID	0,0029781	0,0023852	0,0021891	0,00099681	0,5524529
PARAXANTHINE	0,0003163	0,00036771	0,0001576	0,00016501	0,5524529
PHOSPHOCHOLINE	0,0001289	3,94E-05	9,311E-05	3,82E-05	0,5747405
PICOLINIC ACID	7,97E-06	5,07E-06	5,29E-06	1,45E-06	0,6203031
PYRIDOXAMINE	4,17E-06	3,02E-06	3,85E-06	3,27E-06	0,6205481
QUINATE	3,12E-05	3,38E-05	3,10E-05	2,83E-05	0,644032
RAC-GLYCEROL 1-MYRISTATE	3,502E-05	9,93E-06	2,96E-05	6,05E-06	0,6677892
RETINOATE	1,39E-06	7,81E-07	3,35E-06	4,73E-06	0,6678767
RIBOSE 5-PHOSPHATE	0,0005286	0,00023538	0,0003694	0,00012139	0,6678767
S-(5'-ADENOSYL)-L-HOMOCYSTEINE	1,48E-05	7,64E-06	1,20E-05	4,25E-06	0,6678767
SARCOSINE	0,0005266	0,00016003	0,0003942	9,05E-05	0,6678767
SHIKIMATE	3,67E-06	1,47E-06	2,37E-06	6,52E-07	0,6678767
SN-GLYCERO-3-PHOSPHOCHOLINE	0,0148173	0,00532371	0,0160334	0,00643818	0,7165645
SN-GLYCEROL 3-PHOSPHATE	0,0005759	0,00051698	0,0003302	0,000252	0,7165645
STEARATE	1,38E-05	7,62E-06	9,74E-06	3,00E-06	0,7413628
SUBERIC ACID	0,0061379	0,00282687	0,0041307	0,00059982	0,7664327
SUCCINATE	0,0010274	0,00053957	0,001049	0,00068644	0,7917496
TAURINE	0,0194249	0,00408937	0,0151794	0,00347114	0,7917496
THEOPHYLLINE	0,0002559	0,00029684	0,0002428	0,00028196	0,7917496
Thiamine	5,13E-07	7,16E-07	6,93E-07	1,23E-06	0,7917496
TRANS-ACONITATE	9,88E-06	3,43E-05	0,0001011	0,00042116	0,8125337
TRANS-CINNAMALDEHYDE	4,81E-06	2,31E-06	3,28E-06	1,08E-06	0,8172881
TRANS-CINNAMATE	0,0034775	0,0009822	0,0026841	0,00032495	0,8172881
TRIGONELLINE	0,0001259	0,00011601	9,248E-05	9,38E-05	0,8172881
TRIMETHYLAMINE	3,37E-05	1,32E-05	3,147E-05	9,30E-06	0,843022
URACIL	0,000127	0,00004522	9,956E-05	4,57E-05	0,8949687
URATE	0,000354	0,00015042	0,0003291	0,00014898	0,8949687
URIDINE	0,0013856	0,00053877	0,0011115	0,000627	0,8949687
URIDINE-5-MONOPHOSPHATE	0,000157	8,57E-05	0,000141	0,00003366	0,94737
UROCANATE	0,0001489	0,00011853	9,50E-05	8,29E-05	0,94737
XANTHINE	0,0003009	0,00017625	0,0002736	0,0001738	0,9735145
XANTHOSINE	1,20E-06	6,76E-07	9,55E-07	5,40E-07	0,9736707
XANTHURENIC ACID	2,39E-05	0,00002223	1,47E-05	7,63E-06	0,9736707

Serum samples

	ALS		Control		
	mean	standard error	mean	standard error	raw p-value
10-HYDROXYDECANOATE	0,0010336	0,00039457	0,0009184	0,00071497	0,0060909
1-METHYLADENOSINE	3,153E-05	4,56E-06	3,028E-05	0,00000685	0,0135656
1-OLEOYL-RAC-GLYCEROL	0,0025817	0,00095637	0,0023276	0,00083371	0,0241188
2-AMINO-2-METHYLPROPANOATE	0,0002288	9,15E-05	0,0002227	8,04E-05	0,0241188
2-HYDROXYBUTYRIC ACID	0,0442296	0,01045463	0,0535211	0,01765621	0,0304795
2-HYDROXYPYRIDINE	7,37E-05	9,96E-05	3,449E-05	1,47E-05	0,0354788
3-(2-HYDROXYPHENYL)PROPANOATE	0,0013851	0,00043531	0,0018882	0,00090982	0,0411616
3-AMINO-4-HYDROXYBENZOIC ACID	0,0012551	0,00068451	0,0013245	0,00049327	0,0411616

3-AMINOISOBUTANOATE	0,0012308	0,00029288	0,0013718	0,00062375	0,0511201
3-DEHYDROSHIKIMATE	0,0003955	0,0002391	0,0003807	0,000213	0,0511201
3-METHYGLUTARIC ACID	0,001324	0,00031232	0,0028579	0,00650989	0,0511201
3-Methylhistidine	0,0027384	0,00213251	0,0036443	0,00189194	0,0548586
4-GUANIDINOBUTANOATE	6,93E-05	3,82E-05	6,25E-05	2,74E-05	0,0588224
4-HYDROXY-2-QUINOLINECARBOXYLIC AC	0,0005474	0,00034279	0,0004383	0,00013622	0,0630212
4-METHYL-2-OXO-PENTANOIC ACID	0,0773471	0,01135551	0,0780071	0,01745059	0,0674647
4-METHYL-2-OXO-VALERIC ACID	0,1365614	0,02099466	0,1458267	0,03419622	0,0674647
5,6-DIHYDRO URACIL	0,0006956	7,48E-05	0,0007139	0,0001421	0,0771255
5-OXO-L-PROLINE	0,0001784	3,21E-05	0,0001744	5,07E-05	0,0823629
ADENINE	0,0005363	9,43E-05	0,0004906	0,00014626	0,087885
ADIPIC ACID	0,0002895	0,00005826	0,000336	7,96E-05	0,087885
ALLANTOIN	0,0002733	8,29E-05	0,0002165	5,02E-05	0,0998236
ALPHA-TOCOPHEROL	0,0003119	0,00010303	0,0002861	7,91E-05	0,1062602
AZELAIC ACID	0,0096081	0,00178034	0,0104866	0,00549149	0,1062602
BENZYL ALCOHOL	0,0091518	0,00655118	0,0080587	0,00603334	0,1130215
BETA-ALANINE	1,86E-05	5,93E-06	2,49E-05	1,17E-05	0,1130215
BETAINE	0,1501893	0,02935157	0,1524839	0,03887142	0,1201174
BILIRUBIN	0,0065791	0,00310278	0,0069696	0,00374026	0,1435077
CAFFEATE	0,0003414	4,46E-05	0,0003496	3,69E-05	0,1435077
CATECHOL	0,0004592	0,00052057	0,0002798	0,00020494	0,1799372
CITRAMALATE	0,0007209	0,00027261	0,0005437	0,00021064	0,1799372
CITRULLINE	0,0005596	0,00023804	0,000687	0,00070073	0,1900361
CORTISOL	0,0024404	0,00129256	0,0025322	0,00073049	0,2005465
CREATINE	0,017802	0,00677683	0,0157402	0,00665356	0,2114749
CREATININE	0,4844507	0,04663012	0,4861645	0,07678168	0,2228275
CYTIDINE	8,23E-05	2,20E-05	8,404E-05	4,12E-05	0,2468266
CYTOSINE	0,0004553	8,62E-05	0,000436	7,26E-05	0,2468266
DEOXYCARNITINE	0,0010053	0,00051427	0,0008853	0,00037795	0,2594823
DIETHANOLAMINE	0,0004164	0,00026174	0,0003189	0,00013422	0,2594823
DOPAMINE	0,0001746	3,49E-05	0,0002171	5,03E-05	0,2725804
ELAIDIC ACID	0,0016513	0,00115562	0,0011132	0,00051871	0,2725804
EPINEPHRINE	0,0071364	0,0021587	0,0064933	0,00164743	0,2725804
ETHANOLAMINE	7,24E-05	1,10E-05	7,10E-05	1,18E-05	0,2725804
ETHYLMALONIC ACID	8,38E-05	0,00002106	6,78E-05	2,07E-05	0,2861238
FORMYL-METHIONYL PEPTIDE	0,0003843	8,76E-05	0,0003782	6,68E-05	0,3001148
FUMARATE	0,0005294	0,00013045	0,0004494	0,00008428	0,3001148
GALACTOSAMINE	0,0008528	0,0002867	0,0006842	0,00016126	0,3145545
GAMMA-LINOLENIC ACID	0,0014754	0,0005647	0,0019755	0,00082389	0,3145545
GLUCONIC ACID	0,0016384	0,00049163	0,001358	0,00035428	0,3294436
GLUCURONIC ACID	0,0003325	0,00011144	0,0003441	0,00026158	0,3294436
GLYCERATE	0,0047406	0,00201929	0,0047189	0,00193504	0,3447819
GLYCERO-3-PHOSPHOCHOLINE	0,0098729	0,00458931	0,0170443	0,02650162	0,3447819
GLYCINE	7,29E-05	2,24E-05	9,79E-05	7,84E-05	0,3768005
GUANIDINOACETATE	0,0007738	0,00016346	0,000659	0,00017252	0,3934759
GUANINE	0,001248	0,00022431	0,0012598	0,00020316	0,3934759
GUANOSINE	0,0019067	0,00107959	0,0016032	0,00098642	0,3934759
HIPPURATE	0,0299184	0,02902913	0,0287961	0,02901914	0,4105907
HYPOXANTHINE	0,0091964	0,00404098	0,0085899	0,00280587	0,42814
INDOLE-3-ACETIC ACID	0,0078499	0,00483188	0,0059662	0,00264023	0,42814
INDOXYL SULFATE	0,0359751	0,02262754	0,0365553	0,02014305	0,4461182
INOSINE	0,021219	0,01414736	0,0117617	0,00924096	0,4461182
ISOCITRIC ACID	0,045421	0,01616773	0,0362928	0,01128755	0,4461182
LACTATE	0,4213917	0,03758585	0,42651	0,06465448	0,4461182
L-ALANINE	0,0017948	0,00049751	0,0019703	0,00084244	0,4644923
L-ARGININE	0,0038421	0,0014202	0,005264	0,00594235	0,4833341
L-ASPARAGINE	0,0009727	0,00044495	0,0007701	0,00021506	0,5025556
L-ASPARTATE	0,0004315	0,00019837	0,0004222	0,00012554	0,5221739
LAUROYLCARNITINE	0,000477	0,00049555	0,0004174	0,00021681	0,5221739
L-CARNITINE	0,0727098	0,01439749	0,0744165	0,03339634	0,5221739

LEUCINE	0,0081901	0,00190335	0,0081791	0,00192571	0,5221739
L-GLUTAMIC ACID	0,0039593	0,00118489	0,0044389	0,00156879	0,5221739
L-GLUTAMINE	0,0405196	0,01459512	0,0329964	0,0096063	0,5421785
L-HISTIDINE	0,0052493	0,00091189	0,0066477	0,0039892	0,5421785
LINOLEATE	0,0006997	0,00035497	0,0005483	0,00026836	0,5421785
L-KYNURENINE	0,0002819	0,0001144	0,0002999	0,00012645	0,5421785
L-LYSINE	0,0008193	0,0003436	0,0016641	0,00306102	0,5421785
L-METHIONINE	0,0009616	0,00021875	0,0010046	0,00026697	0,5625582
L-NORVALINE	0,0003875	0,00024644	0,0004319	0,00036105	0,5625582
L-ORNITHINE	0,0001873	6,80E-05	0,0004227	0,00088311	0,5625582
L-PHENYLALANINE	0,0087045	0,00170519	0,0086299	0,00107723	0,5625582
L-PIPECOLIC ACID	0,0008307	0,00071652	0,0006123	0,00021375	0,5625582
L-PROLINE	0,0190599	0,00559921	0,019999	0,00662664	0,6043929
L-SERINE	0,0003307	0,00006836	0,0004214	0,00025701	0,6043929
L-THREONINE	0,0007415	0,00020694	0,0009016	0,00058155	0,6043929
L-TRYPTOPHAN	0,0249461	0,00533643	0,0257032	0,0058257	0,6043929
L-TYROSINE	0,011182	0,00305217	0,011148	0,00182869	0,6043929
LUMICHROME	0,0001971	0,00016652	0,000158	0,0001345	0,6043929
L-VALINE	0,0052863	0,00101146	0,0056029	0,00135197	0,6258208
LYXOSE	0,0016703	0,0002977	0,0017125	0,00028164	0,6258208
MALATE	0,0012963	0,00036207	0,0010562	0,00023452	0,6475694
MANNOSE	0,0091142	0,0067038	0,0110468	0,00771403	0,6475694
METHYL INDOLE-3-ACETATE	0,0056252	0,00426503	0,0049807	0,00409582	0,6475694
METHYL-GUANIDINE	4,88E-05	1,15E-05	5,34E-05	1,54E-05	0,6696231
METHYL-JASMONATE	0,0003283	0,00015166	0,0003183	0,00019196	0,6919654
N,N,N-TRIMETHYL-LYSINE	0,0009475	0,00020382	0,0008685	0,00024795	0,6919654
N6-(DELTA2-ISOPENTENYL)-ADENINE	0,0001989	0,00008174	0,0001518	0,00004329	0,6919654
N-Acetyl-4-O-acetylneuraminic acid	0,0002943	9,54E-05	0,0002595	6,19E-05	0,6919654
N-ACETYL-ALANINE	0,0010114	0,00016646	0,0010915	0,00021259	0,6919654
N-ACETYLGLYCINE	0,0014782	0,0013675	0,0013468	0,00089021	0,7145144
N-ACETYL-METHIONINE	0,0002561	8,25E-05	0,0002323	6,16E-05	0,7145791
N-ACETYL-SERINE	0,0005792	0,00011663	0,0006394	0,000177	0,7145791
N-ACETYL-TRYPTOPHAN	0,0001591	0,00004502	0,0001499	5,12E-05	0,7145791
NICOTINAMIDE	0,0005049	0,00026604	0,0006988	0,00025828	0,7374463
NICOTINATE	0,0002942	0,00005535	0,0002895	4,66E-05	0,7374463
NORLEUCINE	0,0038241	0,00090075	0,0036592	0,00089918	0,7605482
O-ACETYL-CARNITINE	0,0162485	0,0082141	0,0139765	0,00447433	0,7605482
OLEATE	0,0006116	0,00028867	0,0005651	0,00022493	0,7838657
PALMITATE	0,0005367	0,00029417	0,00038	0,00012481	0,7838657
PANTOTHENIC ACID	0,0010121	0,00038371	0,0010061	0,00033841	0,7838657
PARAXANTHINE	0,0025616	0,00166642	0,0033562	0,00288434	0,8073789
PHENYLETHANOLAMINE	0,0001539	1,88E-05	0,0001579	3,42E-05	0,8073789
PIPECOLATE	0,0018699	0,00231978	0,003063	0,00393165	0,8073789
PYRIDOXAMINE	0,0036939	0,00070441	0,0038041	0,00056261	0,8310675
QUINATE	0,0012951	0,00143522	0,0006762	0,0005425	0,8310675
RETINOATE	0,0002293	0,00019832	0,0001666	0,00010456	0,8549105
RETINOL	0,0002994	8,93E-05	0,000335	9,94E-05	0,8549105
RIBOFLAVIN	4,01E-05	6,35E-05	4,78E-05	6,72E-05	0,8788868
SARCOSINE	0,0002898	0,00010977	0,0002603	0,00010764	0,8788868
SEROTONINE	0,0012833	0,00071677	0,0013366	0,00082996	0,8788868
SPERMIDINE	0,0003152	0,00015408	0,0002285	0,00013148	0,9029747
SUBERIC ACID	0,0052097	0,00084556	0,006784	0,00817032	0,9029747
SUCCINATE	0,0060813	0,00111311	0,0055893	0,00138837	0,9029747
TAURINE	0,0075309	0,00412892	0,0076318	0,00214303	0,9271523
THEOBROMINE	0,01454	0,01192705	0,0171095	0,01440866	0,9271523
THEOPHYLLINE	0,0221159	0,01934156	0,0193465	0,01841928	0,9271523
TRANS-ACONITATE	0,0003557	0,00013277	0,0002922	0,000094	0,9513973
TRANS-CINNAMALDEHYDE	0,0001428	3,41E-05	0,0001403	2,91E-05	0,9756874
TYRAMINE	0,0001539	1,88E-05	0,0001579	0,00003416	0,9756874
URACIL	0,0001143	3,47E-05	0,0001186	3,00E-05	0,9756874

URATE	0,098234	0,01583568	0,0975781	0,02184737	0,9756874
URIDINE	0,0074105	0,00228636	0,0078206	0,00182818	1
XANTHINE	0,0055857	0,00148958	0,005904	0,00169298	1

Metabolic profile and pathological alterations in the muscle of patients with early-stage amyotrophic lateral sclerosis.

Débora Lanznaster, Clément Bruno, Jérôme Bourgeais, Patrick Emond, Illyes Zemmoura, Antoine Lefèvre, Pascal Reynier, Sébastien Eymieux, Emmanuelle Blanchard, Patrick Vourc'h, Christian R. Andres, Salah Bakkouche, Olivier Herault, Luc Favard, Philippe Corcia, Hélène Blasco.
Corresponding author: Débora Lanznaster, UMR 1253, iBrain, Université de Tours, INSERM, Tours, France. Email : debora.lanznaster@univ-tours.fr.

Supplementary file S4. Pathway analysis based on the metabolites identified in the muscle and the serum of ALS patients and controls. All the metabolic pathways generated by pathway analysis are listed with the fold enrichment and the p-value.

**Serum**						
**Pathway**	**Total**	**Expected**	**Hits**	**Raw p**	**FDR p**	**Impact**
Biosynthesis of unsaturated fatty acids	36	0.26	2	0.0253	0.981	0
Tyrosine metabolism	42	0.3	2	0.0338	0.981	0.15
Linoleic acid metabolism	5	0.04	1	0.035	0.981	1
Arginine biosynthesis	14	0.1	1	0.0953	1	0
Nicotinate and nicotinamide metabolism	15	0.11	1	0.102	1	0.19
Citrate cycle (TCA cycle)	20	0.14	1	0.134	1	0.03
beta-Alanine metabolism*	21	0.15	1	0.14	1	0
Pyruvate metabolism	22	0.16	1	0.146	1	0
Propanoate metabolism	23	0.16	1	0.152	1	0
Alanine, aspartate and glutamate metabolism*	28	0.2	1	0.182	1	0
Glutathione metabolism*	28	0.2	1	0.182	1	0.01
Arginine and proline metabolism*	38	0.27	1	0.24	1	0.03
Fatty acid elongation	39	0.28	1	0.245	1	0
Fatty acid degradation	39	0.28	1	0.245	1	0
Fatty acid biosynthesis	47	0.33	1	0.288	1	0.01

**commonly altered pathways*

**Muscle**						
**Pathway**	**Total**	**Expected**	**Hits**	**Raw p**	**FDR p**	**Impact**
Glycine, serine and threonine metabolism	33	0.4	4	0.000528	0.0443	0.56
Valine, leucine and isoleucine biosynthesis	8	0.1	2	0.00382	0.16	0
Aminoacyl-tRNA biosynthesis	48	0.59	3	0.0191	0.534	0.17
Glyoxylate and dicarboxylate metabolism	32	0.39	2	0.0568	1	0.15
Valine, leucine and isoleucine degradation	40	0.49	2	0.0842	1	0.02
Taurine and hypotaurine metabolism	8	0.1	1	0.0942	1	0.43
Primary bile acid biosynthesis	46	0.56	2	0.107	1	0.02
Phenylalanine metabolism	10	0.12	1	0.116	1	0
Pantothenate and CoA biosynthesis	19	0.23	1	0.21	1	0.03
Selenocompound metabolism	20	0.25	1	0.22	1	0
Sphingolipid metabolism	21	0.26	1	0.229	1	0
beta-Alanine metabolism*	21	0.26	1	0.229	1	0.1
Alanine, aspartate and glutamate metabolism*	28	0.34	1	0.294	1	0
Glutathione metabolism*	28	0.34	1	0.294	1	0.09
Porphyrin and chlorophyll metabolism	30	0.37	1	0.312	1	0
Cysteine and methionine metabolism	33	0.4	1	0.337	1	0.02
Arginine and proline metabolism*	38	0.47	1	0.378	1	1
Pyrimidine metabolism	39	0.48	1	0.386	1	0.01
Purine metabolism	65	0.8	1	0.559	1	0

**commonly altered pathways*

Metabolic profile and pathological alterations in the muscle of patients with early-stage amyotrophic lateral sclerosis.

Débora Lanznaster, Clément Bruno, Jérôme Bourgeais, Patrick Emond, Illyes Zemmoura, Antoine Lefèvre, Pascal Reynier, Sébastien Eymieux, Emmanuelle Blanchard, Patrick Vourc'h, Christian R. Andres, Salah Bakkouche, Olivier Herault, Luc Favard, Philippe Corcia, Hélène Blasco.
Corresponding author: Débora Lanznaster, UMR 1253, iBrain, Université de Tours, INSERM, Tours, France. Email : debora.lanznaster@univ-tours.fr.

Supplementary file S5. Pathway analysis based on the metabolites identified in the serum and the muscle of ALS patients regarding weight variation. All the metabolic pathways generated by pathway analysis are listed with the fold enrichment and the p-value.

Serum

**Pathway**	**Total**	**Expected**	**Hits**	**Raw p**	**FDR p**	**Impact**
Citrate cycle (TCA cycle)	20	0.19	4	0.0000252	0.00212	0.15
Pyruvate metabolism	22	0.21	3	0.00101	0.0425	0.03
Alanine, aspartate and glutamate metabolism	28	0.27	3	0.00208	0.0582	0.2
Glyoxylate and dicarboxylate metabolism	32	0.31	3	0.00308	0.0646	0,00
Arginine biosynthesis	14	0.14	2	0.00744	0.12	0.12
Butanoate metabolism	15	0.15	2	0.00854	0.12	0,00
Lysine degradation	25	0.24	2	0.0231	0.277	0,00
Linoleic acid metabolism	5	0.05	1	0.0475	0.477	1,00
Nitrogen metabolism	6	0.06	1	0.0568	0.477	0,00
D-Glutamine and D-glutamate metabolism	6	0.06	1	0.0568	0.477	0.5
Aminoacyl-tRNA biosynthesis	48	0.46	2	0.0763	0.583	0,00
Nicotinate and nicotinamide metabolism	15	0.15	1	0.136	0.935	0,00
Histidine metabolism	16	0.15	1	0.145	0.935	0,00
Pantothenate and CoA biosynthesis	19	0.18	1	0.17	1	0.02
beta-Alanine metabolism	21	0.2	1	0.186	1	0.06
Propanoate metabolism	23	0.22	1	0.202	1	0,00
Glycolysis / Gluconeogenesis	26	0.25	1	0.25	1	0,00
Glutathione metabolism	28	0.27	1	0.24	1	0.02
Porphyrin and chlorophyll metabolism	30	0.29	1	0.255	1	0,00
Biosynthesis of unsaturated fatty acids	36	0.35	1	0.298	1	0,00
Arginine and proline metabolism	38	0.37	1	0.312	1	0.09
Pyrimidine metabolism	39	0.38	1	0.319	1	0.03
Tryptophan metabolism	41	0.4	1	0.332	1	0.14
Tyrosine metabolism	42	0.41	1	0.339	1	0.02

Muscle

**Pathway**	**Total**	**Expected**	**Hits**	**Raw p**	**FDR p**	**Impact**
Arginine biosynthesis	14	0.14	3	0.000251	0.0211	0.14
Arginine and proline metabolism	38	0.37	2	0.0503	0.916	0.17
Pyrimidine metabolism	39	0.38	2	0.0527	0.916	0.09
D-Glutamine and D-glutamate metabolism	6	0.06	1	0.0568	0.916	0,00
Nitrogen metabolism	6	0.06	1	0.0568	0.916	0,00
Ascorbate and aldarate metabolism	8	0.08	1	0.075	0.916	0,00
Aminoacyl-tRNA biosynthesis	48	0.46	2	0.0763	0.916	0,00
Nicotinate and nicotinamide metabolism	15	0.15	1	0.136	1	0,00
Histidine metabolism	16	0.15	1	0.145	1	0.12
Galactose metabolism	27	0.26	1	0.233	1	0,00
Glutathione metabolism	28	0.27	1	0.24	1	0,00
Phosphatidylinositol signaling system	28	0.27	1	0.24	1	0.04
Alanine, aspartate and glutamate metabolism	28	0.27	1	0.24	1	0.11
Inositol phosphate metabolism	0	0.29	1	0.255	1	0.13
Glyoxylate and dicarboxylate metabolism	32	0.31	1	0.27	1	0,00
Tyrosine metabolism	42	0.41	1	0.339	1	0.01
Purine metabolism	65	0.63	1	0.476	1	0,00

Metabolic profile and pathological alterations in the muscle of patients with early-stage amyotrophic lateral sclerosis.

Débora Lanznaster, Clément Bruno, Jérôme Bourgeais, Patrick Emond, Illyes Zemmoura, Antoine Lefèvre, Pascal Reynier, Sébastien Eymieux, Emmanuelle Blanchard, Patrick Vourc'h, Christian R. Andres, Salah Bakkouche, Olivier Herault, Luc Favard, Philippe Corcia, Hélène Blasco.
Corresponding author: Débora Lanznaster, UMR 1253, iBrain, Université de Tours, INSERM, Tours, France. Email : debora.lanznaster@univ-tours.fr.

Supplementary file S6. Pathway analysis based on the metabolites identified in the serum and in the muscle of ALS patients regarding disease duration. All the metabolic pathways generated by pathway analysis are listed with the fold enrichment and the p-value.

**Serum**						
**Pathway**	**Total**	**Expected**	**Hits**	**Raw p**	**FDR p**	**Impact**
Riboflavin metabolism	4	0.04	1	0.0382	1	0.50
Pyrimidine metabolism	39	0.38	2	0.0527	1	0.03
Nitrogen metabolism	6	0.06	1	0.0568	1	0
D-Glutamine and D-glutamate metabolism	6	0.06	1	0.0568	1	0.50
Aminoacyl-tRNA biosynthesis	48	0.46	2	0.0763	1	0
Vitamin B6 metabolism	9	0.09	1	0.084	1	0.08
Caffeine metabolism	10	0.1	1	0.0929	1	0
Arginine biosynthesis	14	0.14	1	0.128	1	0.12
Butanoate metabolism	15	0.15	1	0.136	1	0
Histidine metabolism	16	0.15	1	0.145	1	0
Pantothenate and CoA biosynthesis	19	0.18	1	0.17	1	0.02
beta-Alanine metabolism	21	0.2	1	0.186	1	0.06
Glutathione metabolism	28	0.27	1	0.24	1	0.02
Alanine, aspartate and glutamate metabolism	28	0.27	1	0.24	1	0.20
Porphyrin and chlorophyll metabolism	30	0.29	1	0.255	1	0
Glyoxylate and dicarboxylate metabolism	32	0.31	1	0.27	1	0
Glycerophospholipid metabolism	36	0.35	1	0.298	1	0.01
Arginine and proline metabolism	38	0.37	1	0.312	1	0.09
Tryptophan metabolism	41	0.4	1	0.332	1	0.14
Tyrosine metabolism	42	0.41	1	0.339	1	0.02
Purine metabolism	65	0.63	1	0.476	1	0.01

**Muscle**						
**Pathway**	**Total**	**Expected**	**Hits**	**Raw p**	**FDR p**	**Impact**
Taurine and hypotaurine metabolism	8	0.06	2	0.0015	0.126	0.71
Cysteine and methionine metabolism	33	0.26	2	0.0254	1	0.04
Vitamin B6 metabolism	9	0.07	1	0.0677	1	0.08
Biotin metabolism	10	0.08	1	0.075	1	2.00
Caffeine metabolism	10	0.08	1	0.075	1	0.69
Arginine biosynthesis	14	0.11	1	0.104	1	0.06
Ether lipid metabolism	20	0.15	1	0.145	1	0
Lysine degradation	25	0.19	1	0.178	1	0
Glutathione metabolism	28	0.22	1	0.197	1	0
Glycerophospholipid metabolism	36	0.28	1	0.246	1	0.05
Arginine and proline metabolism	38	0.29	1	0.258	1	0.11
Primary bile acid biosynthesis	46	0.36	1	0.304	1	0.01
Aminoacyl-tRNA biosynthesis	48	0.37	1	0.315	1	0
Purine metabolism	65	0.50	1	0.403	1	0.06

Metabolic profile and pathological alterations in the muscle of patients with early-stage amyotrophic lateral sclerosis.
Débora Lanznaster, Clément Bruno, Jérôme Bourgeais, Patrick Emond, Illyes Zemmoura, Antoine Lefèvre, Pascal Reynier, Sébastien Eymieux, Emmanuelle Blanchard, Patrick Vourc'h, Christian R. Andres, Salah Bakkouche, Olivier Herault, Luc Favard, Philippe Corcia, Hélène Blasco.
Corresponding author: Débora Lanznaster, UMR 1253, iBrain, Université de Tours, INSERM, Tours, France. Email : debora.lanznaster@univ-tours.fr.

Supplementary file S7. Correlation between muscle metabolites with levels of *SOD3* and *GLRX2(1)* expression from ALS patients and controls. Significant correlated metabolites are listed together with the Spearman r and the p value.

Correlation of muscle metabolites and *GLRX2(1)* expression

**Controls**	Pearson r	95% confidence interval	P value	Number of XY Pairs
4-METHYL-2-OXO-PENTANOIC_ACID	0,7062	0,3039 to 0,8948	0,0033	15
4-METHYL-2-OXOVALERIC_ACID	0,662	0,2266 to 0,8769	0,0072	15
5-HYDROXYINDOLEACETATE	0,7864	0,4591 to 0,9257	0,0005	15
GUANINE	0,7982	0,4838 to 0,9302	0,0004	15
GUANOSINE	0,6435	0,1957 to 0,8692	0,0096	15
GUANOSINE_5'-MONOPHOSPHATE	0,8697	0,6446 to 0,956	<0,0001	15
HISTAMINE	0,6355	0,1826 to 0,8659	0,0109	15
LACTATE	-0,7428	-0,9091 to -0,3721	0,0015	15
LEUCINE	0,8626	0,6278 to 0,9535	<0,0001	15
L-ISOLEUCINE	0,8389	0,5728 to 0,945	<0,0001	15
L-METHIONINE	0,864	0,6311 to 0,954	<0,0001	15
L-PHENYLALANINE	0,8602	0,622 to 0,9527	<0,0001	15
L-TRYPTOPHAN	0,6086	0,1399 to 0,8545	0,0161	15
L-TYROSINE	0,8954	0,7078 to 0,965	<0,0001	15
L-VALINE	0,661	0,2248 to 0,8765	0,0073	15
LYXOSE	0,831	0,555 to 0,9422	0,0001	15
N-ACETYL-L-METHIONINE	0,8869	0,6866 to 0,9621	<0,0001	15
N-ACETYLNEURAMINATE	0,5225	0,01392 to 0,8163	0,0457	15
RIBOSE_5-PHOSPHATE	0,5999	0,1265 to 0,8507	0,0181	15
TRIMETHYLAMINE	0,6577	0,2193 to 0,8751	0,0077	15
URIDINE	0,703	0,2981 to 0,8935	0,0035	15
XANTHINE	0,8044	0,4968 to 0,9324	0,0003	15
XANTHOSINE	0,6096	0,1415 to 0,8549	0,0158	15

**ALS**	Pearson r	95% confidence interval	P value	Number of XY Pairs
GUANIDINOACETATE	-0,648	-0,9074 to -0,03099	0,0428	10

Correlation of muscle metabolites and *SOD3* expression

**Control**	Pearson r	95% confidence interval	P value	Number of XY Pair
2-HYDROXYPYRIDINE	-0,5192	-0,8148 to -0,009483	0,0473	15
5-HYDROXYLYSINE	0,5742	0,08778 to 0,8395	0,0252	15
ADENOSINE_5'-MONOPHOSPHATE	-0,6091	-0,8547 to -0,1408	0,0159	15
C10-CARNITINE	0,6026	0,1307 to 0,8519	0,0174	15
C4-CARNITINE	0,6817	0,2604 to 0,8849	0,0051	15
C8-CARNITINE	0,6118	0,145 to 0,8559	0,0154	15
CYTIDINE	-0,5149	-0,8128 to -0,003601	0,0495	15
EPINEPHRINE	0,5441	0,04416 to 0,8261	0,036	15
ETHANOLAMINE_PHOSPHATE	-0,5696	-0,8375 to -0,08095	0,0267	15
HIPPURATE	-0,6049	-0,8529 to -0,1343	0,0169	15
HYPOXANTHINE	-0,5709	-0,8381 to -0,08289	0,0262	15
INOSINE	-0,5149	-0,8128 to -0,00362	0,0495	15
L-CARNITINE	0,6061	0,1361 to 0,8534	0,0166	15
L-GLUTAMIC_ACID	0,5457	0,04641 to 0,8268	0,0354	15
SUCCINATE	0,6995	0,2919 to 0,8921	0,0037	15
URATE	-0,653	-0,8732 to -0,2115	0,0083	15
XANTHURENIC_ACID	-0,6771	-0,8831 to -0,2524	0,0056	15

**ALS**	Pearson r	95% confidence interval	P value	Number of XY Pair

Cyclic_AMP	0,7674	0,2666 to 0,9419	0,0096	10
DEOXYCARNITINE	0,6727	0,07473 to 0,9148	0,0331	10
L-HISTIDINE	0,6478	0,03073 to 0,9074	0,0428	10
L-ISOLEUCINE	-0,7209	-0,9289 to -0,1672	0,0186	10
MANNOSE	0,7162	0,1577 to 0,9276	0,0198	10
THEOPHYLLINE	0,705	0,1354 to 0,9243	0,0228	10

s

s

Metabolic profile and pathological alterations in the muscle of patients with early-stage amyotrophic lateral sclerosis.
Débora Lanznaster, Clément Bruno, Jérôme Bourgeais, Patrick Emond, Illyes Zemmoura, Antoine Lefèvre, Pascal Reynier, Sébastien Eymieux, Emmanuelle Blanchard, Patrick Vourc'h, Christian R. Andres, Salah Bakkouche, Olivier Herault, Luc Favard, Philippe Corcia, Hélène Blasco.
Corresponding author: Débora Lanznaster, UMR 1253, iBrain, Université de Tours, INSERM, Tours, France. Email : debora.lanznaster@univ-tours.fr.

Supplementary file S8. Correlation between metabolites from muscle or serum with LDH activity or the ratio complex II/CS from ALS patients and controls. Significant correlated metabolites are listed together with the Spearman r and the p value.

LDH x muscle metabolites

Controls

	Spearman r	95% confidence interval	P value	Number of XY Pairs
3-UREIDOPROPIONATE	0,4923	0,01802 to 0,7857	0,038	18
CITRULLINE	0,6223	0,2048 to 0,8482	0,0058	18
DEOXYCARNITINE	0,5955	0,1636 to 0,8358	0,0091	18
ETHYLMALONIC_ACID	-0,6639	-0,867 to -0,2718	0,0027	18
L-KYNURENINE	-0,5046	-0,7919 to -0,03448	0,0327	18
L-PROLINE	0,548	0,0942 to 0,8132	0,0186	18

ALS

	Spearman r	95% confidence interval	P value	Number of XY Pairs
2-DEOXY-GLUCOSE	0,5417	0,0671 to 0,8164	0,0267	17
3-SULFINO-L-ALANINE	-0,5172	-0,8047 to -0,03313	0,0355	17
4-AMINOBUTANOATE	0,6609	0,2498 to 0,8702	0,0048	17
ADENOSINE_5'-MONOPHOSPHATE	-0,5359	-0,8136 to -0,05897	0,029	17
ALPHA-GLUCOSE_1-PHOSPHATE	-0,5245	-0,8082 to -0,04321	0,0327	17
C16-CARNITINE	0,488	-0,005818 to 0,7905	0,0485	17
C18-CARNITINE	0,4902	-0,00299 to 0,7916	0,0478	17
C2-CARNITINE	0,5515	0,08101 to 0,821	0,0237	17
C3-CARNITINE	0,7157	0,3447 to 0,8933	0,0017	17
C4-CARNITINE	0,7059	0,3271 to 0,8892	0,0021	17
CHOLINE	0,5763	0,1171 to 0,8325	0,0171	17
CREATININE	0,5123	0,02647 to 0,8023	0,0376	17
GLUTATHIONE	0,4902	-0,00299 to 0,7916	0,0478	17
HIPPURATE	0,5025	0,01327 to 0,7976	0,0419	17
LACTATE	0,4975	0,006735 to 0,7952	0,0442	17
L-PIPECOLIC_ACID	0,5411	0,06631 to 0,8161	0,0267	17
O-ACETYL-L-CARNITINE	0,5074	0,01985 to 0,7999	0,0397	17
PANTOTHENIC_ACID	0,5637	0,09865 to 0,8267	0,0203	17
S-(5'-ADENOSYL)-L-HOMOCYSTEINE	0,6638	0,2546 to 0,8714	0,0046	17
TRIMETHYLAMINE	0,598	0,1496 to 0,8424	0,0128	17

LDH x serum metabolites

Controls

	Spearman r	95% confidence interval	P value	Number of XY Pairs
4-METHYL-2-OXOVALERIC_ACID	0,5656	0,1505 to 0,8112	0,0093	20
5-HYDROXYINDOLEACETATE	0,4883	0,04443 to 0,7712	0,0289	20
DEOXYCARNITINE	0,4528	-0,001195 to 0,752	0,045	20
L-ANSERINE	0,6211	0,2329 to 0,8385	0,0035	20

ALS

	Spearman r	95% confidence interval	P value	Number of XY Pairs
Methylhistidine	0,5347	0,05737 to 0,8131	0,0288	17
HIPPURATE	0,5436	0,06977 to 0,8173	0,0259	17
L-ORNITHINE	0,4926	0,0002416 to 0,7928	0,0465	17
MANNOSE	-0,5441	-0,8175 to -0,07056	0,0259	17
METHYL_INDOLE-3-ACETATE	0,5858	0,1312 to 0,8368	0,0152	17
N6-(DELTA2-ISOPENTENYL)-ADENINE	-0,5224	-0,8072 to -0,04028	0,0332	17
SUBERIC_ACID	-0,5383	-0,8148 to -0,0624	0,0276	17

complex II/CS x muscle metabolites

Controls				
	Spearman r	95% confidence interval	P value	Number of XY Pairs
MANNOSE	0,686	0,3089 to 0,8767	0,0017	18
PARAXANTHINE	0,6629	0,2701 to 0,8665	0,0027	18

ALS				
Metabolites	Spearman r	95% confidence interval	P value	Number of XY Pairs
3-AMINO-4-HYDROXYBENZOIC_ACID	0,6066	0,1628 to 0,8463	0,0112	17
3-HYDROXYBUTANOIC_ACID	-0,4926	-0,7928 to -0,0002197	0,0462	17
4-METHYL-2-OXO-PENTANOIC_ACID	-0,5209	-0,8065 to -0,03823	0,0338	17
C5-CARNITINE	-0,5946	-0,8408 to -0,1444	0,0133	17
HISTAMINE	0,5344	0,05691 to 0,8129	0,0289	17
SN-GLYCEROL_3-PHOSPHATE	-0,4926	-0,7928 to -0,0002197	0,0462	17

complex II/CS x serum metabolites

Controls				
	Spearman r	95% confidence interval	P value	Number of XY Pairs
4-HYDROXY-L-PROLINE	0,6916	0,3021 to 0,8833	0,0028	17
5-HYDROXYINDOLEACETATE	0,5605	0,09404 to 0,8252	0,0209	17
Cyclic_AMP	0,4853	-0,009467 to 0,7891	0,05	17
FORMYL-L-METHIONYL_PEPTIDE	0,5221	0,03991 to 0,8071	0,0333	17
GLYCERATE	-0,5126	-0,8025 to -0,02694	0,037	17
GLYCOLALDEHYDE_DIMER	0,5	0,01 to 0,7964	0,0427	17
INOSINE_5'-MONOPHOSPHATE	0,5762	0,1169 to 0,8324	0,0171	17
LACTATE	0,5602	0,09354 to 0,825	0,0211	17
L-ANSERINE	0,6167	0,1784 to 0,8508	0,0097	17
L-ARABITOL	0,5528	0,08294 to 0,8216	0,0231	17
L-GLUTAMINE	0,6376	0,2116 to 0,86	0,007	17
LYXOSE	-0,586	-0,8369 to -0,1315	0,015	17

ALS				
	Spearman r	95% confidence interval	P value	Number of XY Pairs
1-METHYLADENOSINE	-0,6042	-0,8452 to -0,159	0,0116	17
3-AMINO-4-HYDROXYBENZOIC_ACID	0,4886	-0,005049 to 0,7908	0,0481	17
ALLANTOIN	0,5921	0,1407 to 0,8397	0,0138	17
CORTISOL	-0,5307	-0,8112 to -0,05178	0,0302	17
EPINEPHRINE	-0,527	-0,8094 to -0,04668	0,0315	17
FUMARATE	-0,5504	-0,8205 to -0,07944	0,0238	17
GALACTOSAMINE	-0,5516	-0,821 to -0,08119	0,0234	17
GLYCERATE	0,5925	0,1413 to 0,8399	0,0136	17
L-ALANINE	-0,6177	-0,8512 to -0,18	0,0095	17
LAUROYLCARNITINE	-0,5074	-0,8 to -0,01987	0,0394	17
L-PROLINE	-0,5577	-0,8239 to -0,09	0,0217	17
MALATE	-0,5012	-0,797 to -0,01164	0,0421	17
METHYL-GUANIDINE	0,5467	0,0742 to 0,8187	0,0249	17
OLEATE	-0,5823	-0,8353 to -0,126	0,0158	17
RIBOFLAVIN	0,5	0,01 to 0,7964	0,0427	17
TRANS-CINNAMALDEHYDE	-0,6847	-0,8803 to -0,29	0,0031	17

Metabolic profile and pathological alterations in the muscle of patients with early-stage amyotrophic lateral sclerosis.

Débora Lanznaster*, Clément Bruno*, Jérôme Bourgeais, Patrick Emond, Ilyess Zemmoura, Antoine Lefèvre, Pascal Reynier, Sébastien Eymieux, Emmanuelle Blanchard, Patrick Vourc'h, Christian R. Andres, Salah Bakkouche, Olivier Herault, Luc Favard, Philippe Corcia, Hélène Blasco. *equal author contribution

Corresponding author: Débora Lanznaster, PhD; Email: debora.lanznaster@univ-tours.fr

Supplementary figure S1. Metabolomics findings from serum and clinical parameters of ALS at diagnosis. **A)** Score scatter plot based on the PLS-DA models from serum to explain ALSFRS-r (p-CV-ANOVA=0.004). **B**) Loading scatter plot presenting the top 15 metabolites identified in serum by the PLS-DA model to explain ALSFRS-r at diagnosis. **C)** Score scatter plot based on the PLS-DA models from serum to explain the age at onset (p-CV-ANOVA=0.05). **D)** Loading scatter plot presenting the top 15 metabolites identified in serum by the PLS-DA model to explain age at onset.

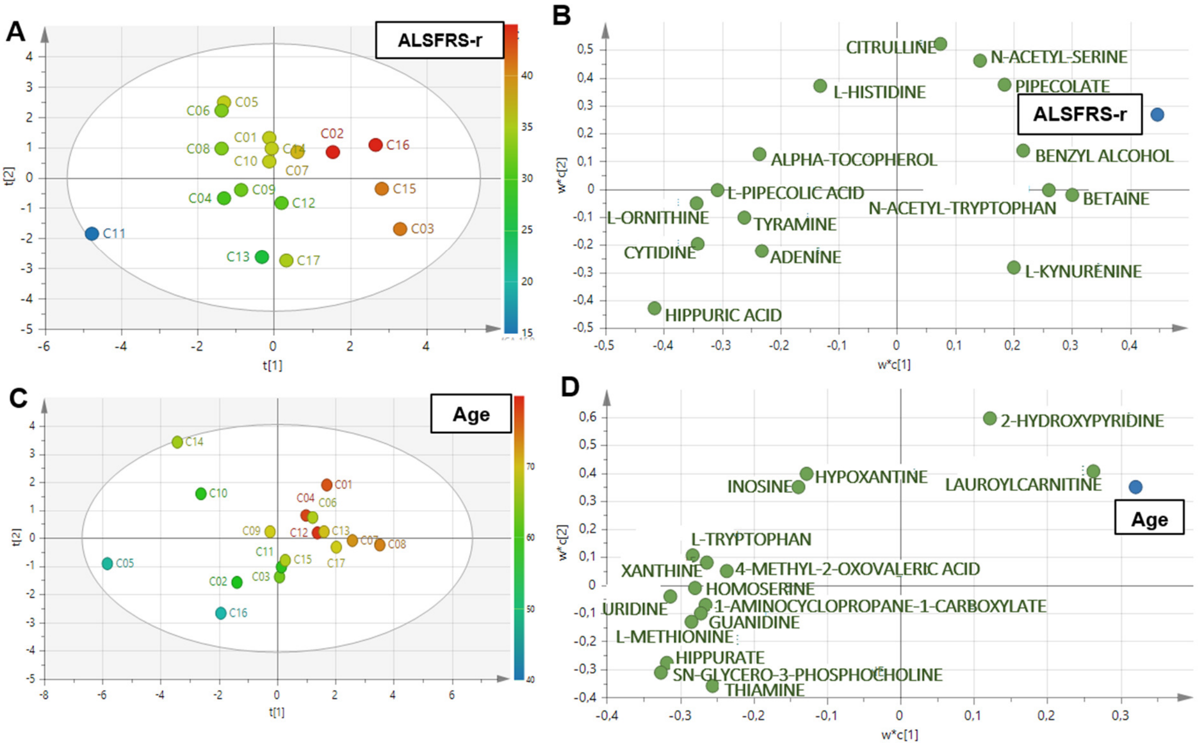

Metabolic profile and pathological alterations in the muscle of patients with early-stage amyotrophic lateral sclerosis.

Débora Lanznaster*, Clément Bruno*, Jérôme Bourgeais, Patrick Emond, Ilyess Zemmoura, Antoine Lefèvre, Pascal Reynier, Sébastien Eymieux, Emmanuelle Blanchard, Patrick Vourc'h, Christian R. Andres, Salah Bakkouche, Olivier Herault, Luc Favard, Philippe Corcia, Hélène Blasco. *equal author contribution

Corresponding author: Débora Lanznaster, PhD; Email: debora.lanznaster@univ-tours.fr

Supplementary Figure S2. Multivariate analysis of the metabolome profile from serum of ALS patients and the variation of weight over 9 months (A-C) and disease duration (D-E). A) Score scatter plot based on the PLS-DA models from serum to explain the weight variation, with R2X=0.66, R2Y=0.917, Q2=0.695 and p=0.07 for the CV ANOVA; the color scale represents the weight variation (gain towards red, loss towards blue) over 9 months. B) Loading scatter plot presenting the top 15 metabolites identified by the PLS-DA. The seven metabolites that had VIP score higher than 1 are written in blue. C) Pathway analysis with the 15 VIP metabolites highlighted alterations in the TCA cycle ($p<0.001$); pyruvate metabolism (p=0.001); alanine, aspartate and glutamate metabolism (p=0.002); glyoxylate and dicarboxylate metabolism (p=0.003); arginine biosynthesis (p=0.007); butanoate metabolism (p=0.008); lysine degradation (p=0.0231) and linoleic acid metabolism (p=0.0475). Each node represents a metabolite set with its color based on its p-value and its size is based on the pathway impact. The complete list of metabolic pathways is described in Supplementary file 5. D) Score scatter plot based on the PLS-DA models from serum to explain the weight variation, with R2X=0.599, R2Y=0.882, Q2=0.757 and p=0.0008 for the CV-ANOVA test; the color scale represents the disease duration (long towards red, short towards blue). E) Loading scatter plot presenting the top 15 metabolites identified by the PLS-DA. The four metabolites that had VIP score higher than 1 are written in blue.

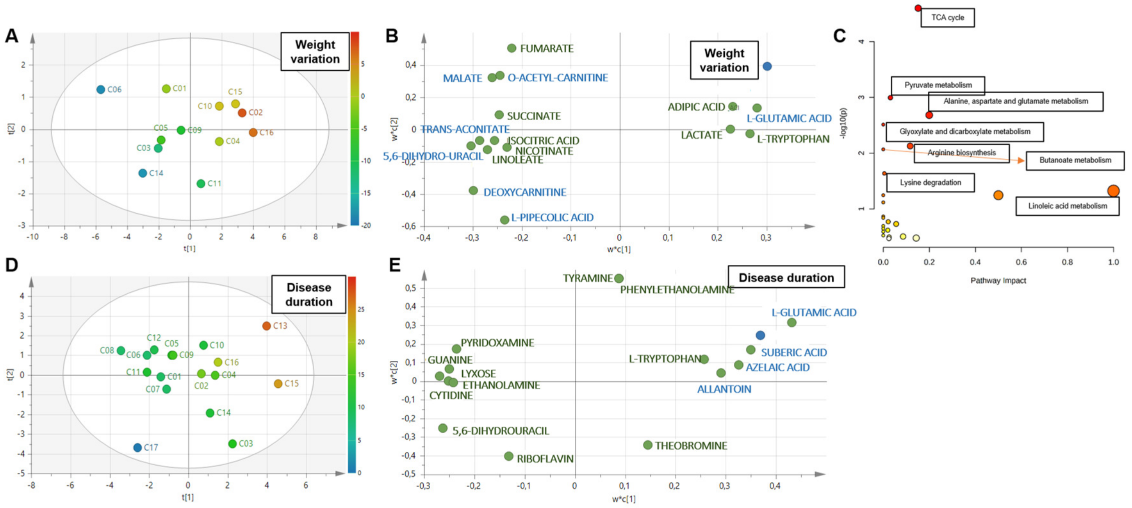

Metabolic profile and pathological alterations in the muscle of patients with early-stage amyotrophic lateral sclerosis.

Débora Lanznaster*, Clément Bruno*, Jérôme Bourgeais, Patrick Emond, Ilyess Zemmoura, Antoine Lefèvre, Pascal Reynier, Sébastien Eymieux, Emmanuelle Blanchard, Patrick Vourc'h, Christian R. Andres, Salah Bakkouche, Olivier Herault, Luc Favard, Philippe Corcia, Hélène Blasco. *equal author contribution

Corresponding author: Débora Lanznaster, PhD; Email: debora.lanznaster@univ-tours.fr

Supplementary figure S3. Metabolomics findings in muscle and clinical parameters of ALS at diagnosis. **A)** Score scatter plot based on the OPLS-DA models from muscle to explain the site at onset (p-CV-ANOVA=0.017) (blue: spinal onset; green: bulbar onset). **B**) Loading scatter plot presenting the top 15 metabolites identified by the OPLS-DA to explain the site of onset. **C)** Score scatter plot based on the PLS-DA models from muscle to explain the FVC (p-CV-ANOVA=0.036). The color scale represents the FVC (high towards red, low towards blue). **D)** Loading scatter plot presenting the top 15 metabolites identified by the PLS-DA model to explain FVC at diagnosis. **E)** Score scatter plot based on the PLS-DA models from muscle to explain the weight (p-CV-ANOVA=0.04). The color scale represents the weight at diagnosis (high towards red, low towards blue). **F)** Loading scatter plot presenting the top 15 metabolites identified by the PLS-DA model to explain weight at diagnosis. **G)** Score scatter plot based on the PLS-DA models from muscle to explain the age at onset (p-CV-ANOVA=0.004). The color scale represents the age at onset (old towards red, young towards blue). **H)** Loading scatter plot presenting the top 15 metabolites identified by the PLS-DA model to explain age at onset.

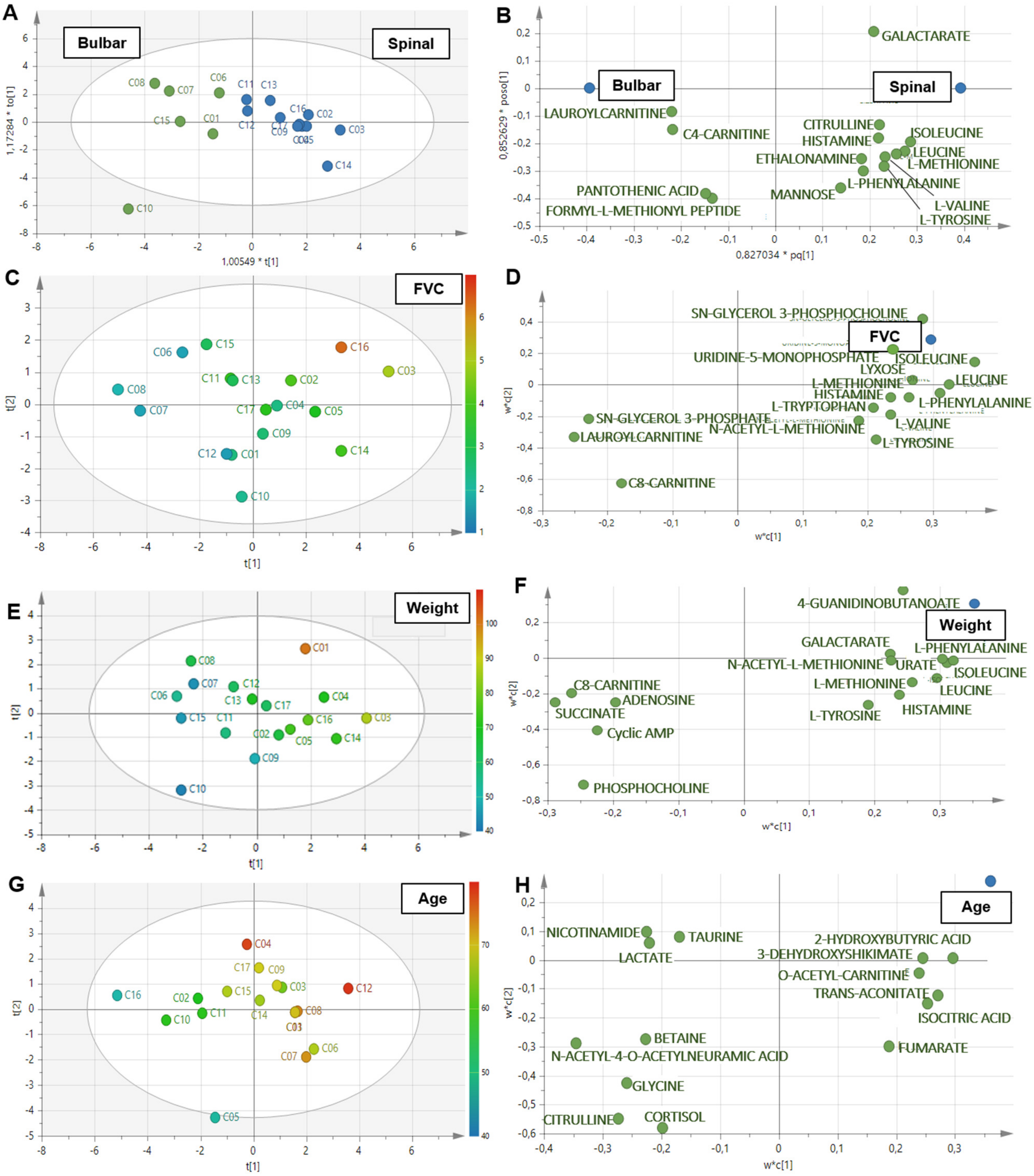
A
Bulbar
Spinal
C08
C07
C06
C11
C13
C15
C01
C12
C16
C02
C09
C03
C14
C10
1,17284 * to[1]
1,00549 * t[1]
B
GALACTARATE
Bulbar
Spinal
LAUROYLCARNITINE
C4-CARNITINE
CITRULLINE
HISTAMINE
ISOLEUCINE
LEUCINE
L-METHIONINE
ETHALONAMINE
L-PHENYLALANINE
PANTOTHENIC ACID
MANNOSE
FORMYL-L-METHIONYL PEPTIDE
L-VALINE
L-TYROSINE
0,852629 * poso[1]
0,827034 * pq[1]
C
FVC
C15
C16
C06
C03
C08
C11
C13
C02
C07
C17
C04
C05
C09
C12
C01
C14
C10
t[2]
t[1]
D
SN-GLYCEROL 3-PHOSPHOCHOLINE
FVC
URIDINE-5-MONOPHOSPHATE
ISOLEUCINE
LYXOSE
L-METHIONINE
LEUCINE
HISTAMINE
L-PHENYLALANINE
L-TRYPTOPHAN
SN-GLYCEROL 3-PHOSPHATE
N-ACETYL-L-METHIONINE
L-VALINE
LAUROYLCARNITINE
L-TYROSINE
C8-CARNITINE
w*c[2]
w*c[1]
E
Weight
C01
C08
C07
C12
C06
C13
C17
C04
C15
C11
C16
C03
C02
C05
C14
C09
C10
t[2]
t[1]
F
4-GUANIDINOBUTANOATE
Weight
GALACTARATE
L-PHENYLALANINE
N-ACETYL-L-METHIONINE
URATE
ISOLEUCINE
C8-CARNITINE
L-METHIONINE
LEUCINE
ADENOSINE
SUCCINATE
L-TYROSINE
HISTAMINE
Cyclic AMP
PHOSPHOCHOLINE
w*c[2]
w*c[1]
G
Age
C04
C17
C09
C03
C12
C16
C02
C15
C10
C11
C14
C08
C06
C07
C05
t[2]
t[1]
H
Age
NICOTINAMIDE
TAURINE
2-HYDROXYBUTYRIC ACID
LACTATE
3-DEHYDROXYSHIKIMATE
O-ACETYL-CARNITINE
TRANS-ACONITATE
ISOCITRIC ACID
BETAINE
FUMARATE
N-ACETYL-4-O-ACETYLNEURAMIC ACID
GLYCINE
CITRULLINE
CORTISOL
w*c[2]
w*c[1]

Metabolic profile and pathological alterations in the muscle of patients with early-stage amyotrophic lateral sclerosis.

Débora Lanznaster*, Clément Bruno*, Jérôme Bourgeais, Patrick Emond, Ilyess Zemmoura, Antoine Lefèvre, Pascal Reynier, Sébastien Eymieux, Emmanuelle Blanchard, Patrick Vourc'h, Christian R. Andres, Salah Bakkouche, Olivier Herault, Luc Favard, Philippe Corcia, Hélène Blasco. *equal author contribution

Corresponding author: Débora Lanznaster, PhD; Email: debora.lanznaster@univ-tours.fr

Supplementary Figure S4. Multivariate analysis of the metabolome profile muscle of ALS patients and the variation of weight over 9 months (A-C) and disease duration (D-E). A) Score scatter plot based on the PLS-DA models from muscle to explain the weight variation, with R2X=0.601, R2Y=0.924, Q2=0.766 and p=0.04 for the CV-ANOVA test; the color scale represents the weight variation (gain towards red, loss towards blue) over 9 months. B) Loading scatter plot presenting the top 15 metabolites identified by the PLS-DA. The six metabolites that had VIP score higher than 1 are written in blue. C) Pathway analysis with the 15 VIP metabolites highlighted alterations in arginine biosynthesis ($p<0.001$); arginine and proline metabolism ($p=0.0503$); and pyrimidine metabolism ($p=0.0527$). The complete list of metabolic pathways is described in Supplementary file 5. D) Score scatter plot based on the PLS-DA models from muscle to explain the disease duration, with R2X=0.472, R2Y=0.9, Q2=0.492 and p=12 for the CV-ANOVA test; the color scale represents the disease duration (long towards red, short towards blue). E) Loading scatter plot presenting the top 15 metabolites identified by the PLS-DA. The six metabolites that had VIP score higher than 1 are written in blue.

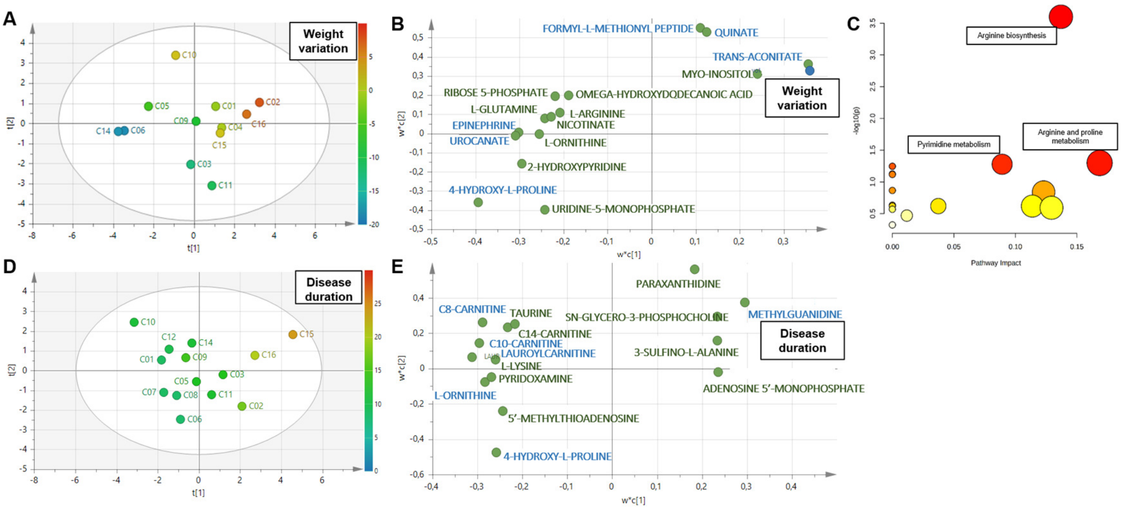

Metabolic profile and pathological alterations in the muscle of patients with early-stage amyotrophic lateral sclerosis.

Débora Lanznaster*, Clément Bruno*, Jérôme Bourgeais, Patrick Emond, Ilyess Zemmoura, Antoine Lefèvre, Pascal Reynier, Sébastien Eymieux, Emmanuelle Blanchard, Patrick Vourc'h, Christian R. Andres, Salah Bakkouche, Olivier Herault, Luc Favard, Philippe Corcia, Hélène Blasco. *equal author contribution

Corresponding author: Débora Lanznaster, PhD; Email: debora.lanznaster@univ-tours.fr

Supplementary Figure S5. Analysis of the most discriminant metabolites from both serum and muscle of ALS patients. Venn diagrams drawn between serum and muscle revealed that lauroylcarnitine was common to models explaining site at onset (A) and FVC (B). Correlation of lauroylcarnitine levels with FVC at diagnosis revealed that high levels of this metabolite correlated with low FVC in serum (C; Pearson r: -0.4291, 95%CI: -0.76;0.08; R2: 0.18; p=0.0972) and specially in muscle (D; Pearson r: -0.5311, 95%CI: -0.81; -0.07; R2: 0.28; p=0.0283). Dotted lines represent the 95%CI. F-G) Venn diagram analysis performed with the 15 metabolites with the highest VIP used to explain disease progression (variation of weight, ALSFRS-r over 9 months and disease duration) from serum (E) or muscle (F) metabolome. Venn diagrams build with Venny 2.1.0 (https://bioinfogp.cnb.csic.es/tools/venny/index.html).

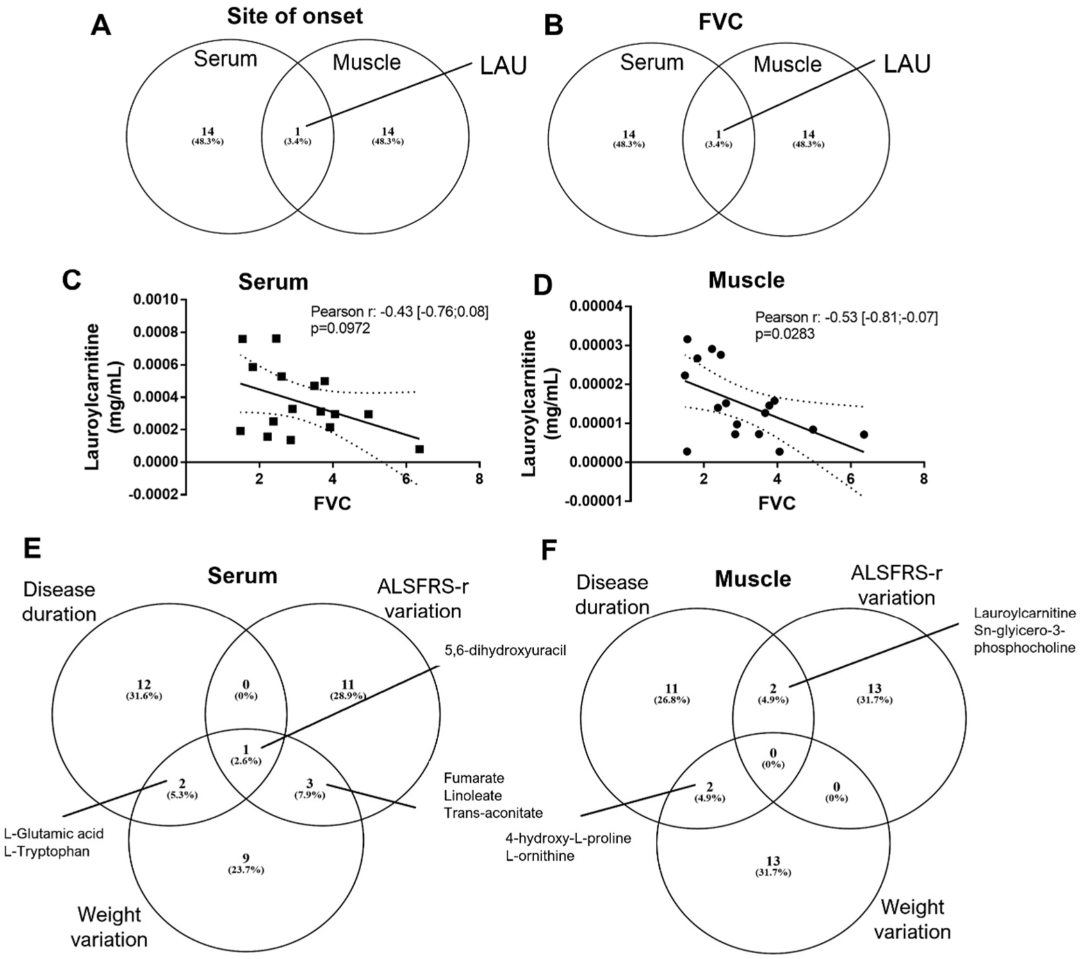
A
Site of onset
Serum
Muscle
LAU
14
(48.3%)
1
(3.4%)
14
(48.3%)
B
FVC
Serum
Muscle
LAU
14
(48.3%)
1
(3.4%)
14
(48.3%)
C
Serum
Pearson r: -0.43 [-0.76;0.08]
p=0.0972
Lauroylcarnitine (mg/mL)
FVC
D
Muscle
Pearson r: -0.53 [-0.81;-0.07]
p=0.0283
Lauroylcarnitine (mg/mL)
FVC
E
Serum
Disease duration
ALSFRS-r variation
Weight variation
12
(31.6%)
0
(0%)
11
(28.9%)
1
(2.6%)
2
(5.3%)
3
(7.9%)
9
(23.7%)
5,6-dihydroxyuracil
Fumarate
Linoleate
Trans-aconitate
L-Glutamic acid
L-Tryptophan
F
Muscle
Disease duration
ALSFRS-r variation
Weight variation
11
(26.8%)
2
(4.9%)
13
(31.7%)
0
(0%)
2
(4.9%)
0
(0%)
13
(31.7%)
Lauroylcarnitine
Sn-glyicero-3-phosphocholine
4-hydroxy-L-proline
L-ornithine
